# Deep-learning endomicroscope with large field-of-view and depth-of-field for real-time in vivo imaging of epithelial cancer hallmarks

**DOI:** 10.1073/pnas.2602705123

**Published:** 2026-05-11

**Authors:** Huayu Hou, Jimin Wu, Jinyun Liu, Vivek Boominathan, Argaja Shende, Karthik Goli, Jennifer Carns, Richard A. Schwarz, Ann M. Gillenwater, Preetha Ramalingam, Mila P. Salcedo, Kathleen M. Schmeler, Tomasz S. Tkaczyk, Jacob T. Robinson, Ashok Veeraraghavan, Rebecca R. Richards-Kortum

**Affiliations:** ^a^Department of Bioengineering, Rice University, Houston, TX 77005; ^b^Department of Electrical and Computer Engineering, Rice University, Houston, TX 77005; ^c^Department of Head and Neck Surgery, The University of Texas MD Anderson Cancer Center, Houston, TX 77030; ^d^Department of Pathology, The University of Texas MD Anderson Cancer Center, Houston, TX 77030; ^e^Department of Gynecologic Oncology and Reproductive Medicine, The University of Texas MD Anderson Cancer Center, Houston, TX 77030; ^f^Department of Neuroscience, Baylor College of Medicine, Houston, TX 77030; ^g^Department of Computer Science, Rice University, Houston, TX 77005

**Keywords:** deep learning, endomicroscopy, extended depth-of-field, large field-of-view, in vivo imaging

## Abstract

In vivo microscopy (IVM) has shown great promise to improve early detection of epithelial precancer, but it suffers from fundamental trade-offs that limit the resolution, field-of-view (FOV) and depth-of-field (DOF). Here, we present PrecisionView, a compact, deep learning-enabled endomicroscope that breaks these constraints and achieves 20 mm^2^ FOV and 500 µm DOF with 4 µm resolution, representing approximately 5× increase in FOV and 8× larger DOF compared to conventional IVM with similar resolution. PrecisionView integrates a deep learning-optimized phase mask and real-time reconstruction, enabling rapid in vivo assessment of two key hallmarks of cancer: epithelial cell nuclear morphology and subsurface microvasculature through fluorescence and reflectance imaging. By imaging the oral cavity of healthy volunteers and cervical specimens with precancerous lesions, PrecisionView generates large-scale (1 to 3 cm^2^) coregistered maps of cellular and vascular structures, revealing distinct microscopic patterns associated with anatomic structures and precancerous lesions. Our results suggest the potential of this computational endomicroscope to address the unmet need for early cancer detection at the point of care.

## Significance

Early cancer detection is crucial for improving patient survival, yet current diagnostic tools remain limited in their ability to comprehensively assess large, heterogeneous lesions at the cellular level in vivo. We introduce a compact and affordable AI-powered endomicroscope (PrecisionView) that overcomes fundamental trade-offs in resolution, field-of-view, and depth-of-field that constrain existing in vivo microscopy technologies. By enabling real-time, wide-area visualization of cellular morphology and microvasculature at the point of care, PrecisionView reveals focal microscopic features associated with anatomic structures or pathological abnormalities across extensive epithelial regions. PrecisionView has the potential to improve early cancer detection and expand access to high-quality cancer detection in both high- and low-resource clinical settings.

Cancer is a leading cause of premature death worldwide (1, 2), and epithelial cancers account for 80 to 90% of all cancer cases (3). Most epithelial cancer patients are diagnosed with late-stage disease due to a lack of effective diagnostic methods, resulting in low overall 5-y survival rates (4, 5). Histopathologic examination of tissue biopsy is the gold standard to diagnose dysplasia and cancer. However, biopsy is an invasive procedure and requires substantial infrastructure to prepare and interpret histology slides. In the case of large, heterogeneous lesions, only limited tissue areas can be assessed with biopsy. Because most suspicious lesions are benign (6, 7), it is extremely challenging even for experts to determine where and when to perform invasive biopsy (8, 9). As a result, the accuracy of pathologic diagnosis suffers from sampling error (10, 11).

In vivo microscopy (IVM) has shown promise to improve the early detection of epithelial cancers (11–13). The morphological hallmarks of cancer, including alterations in neoplastic cell nuclei and supporting microvasculature, provide predictive hallmarks of precancer (9, 14–20). Simultaneous assessment of changes in both cell nuclei and microvasculature can potentially improve accuracy for early cancer and precancer detection. Pilot studies have demonstrated promising clinical performance of IVM for a variety of cancer types (9, 18–20). However, clinical translation of IVM is hindered by fundamental limitations of conventional optics. The field-of-view (FOV) of current IVM is too small (usually <0.5 mm^2^) relative to typical size (~cm^2^) of suspicious lesions (19–32). The scale-dependent geometric aberrations from miniature optics (33) or the small size of image relay probes (20, 21, 23–25, 30) fundamentally restrict the FOV of imaging systems. It is difficult to fully examine large, heterogeneous lesions at risk with current IVM to identify which regions may harbor focal neoplasia (11). Expanding the FOV with conventional approaches dramatically increases the system form factor and complexity, reducing clinical usability. Integrating lateral scanning and image mosaicking provides an alternative approach to overcoming FOV limitations. However, the limited capture area per image acquisition makes it challenging to maintain sufficient overlap during in vivo tissue imaging. As a result, the total mosaicked imaging field is usually restricted to less than 10 mm^2^, with missing regions caused by gaps in the imaging path (21, 34, 35).

Another challenge is that the depth-of-field (DOF) of current systems is inherently coupled to the numerical aperture (NA) and therefore spatial resolution. To achieve cellular resolution for evaluation of microscopic features (<4 µm), conventional microscopes typically have a DOF less than 60 µm (33, 36, 37). This limited DOF is insufficient to accommodate irregularities in the tissue surface, which can be up 200 µm (36–38), resulting in partially out-of-focus images and loss of critical microscopic information. This issue is more significant when imaging across a large tissue area with a large-FOV device. Moreover, current IVM with limited DOF is not capable of simultaneously capturing in-focus images of both cell nuclei and microvasculature. While cell nuclei are best visualized at the epithelial surface, supporting microvasculature is located 100 to 200 µm deeper (20, 21). The depth of microvasculature also varies over a large depth range of several hundred of microns (20). Extended DOF imaging is traditionally achieved through focus stacking, in which multiple images acquired at different focal planes are computationally fused to generate an all-in-focus reconstruction (39–41). Addressing axial variation with conventional systems therefore typically requires an additional axial scanning module to continuously adjust the focal plane, which significantly increases cost and complexity, and reduces clinical usability.

Computational imaging technologies and deep learning have emerged as powerful tools to overcome the limitations of conventional optics. The integration of wavefront encoding with computational algorithms has proven to be a promising approach for improving system capabilities beyond those of conventional microscopy, enabling extended DOF (36, 37, 42–44), aberration correction (45–47), single-shot 3D imaging (48, 49), and lensless imaging (50–53). Moreover, recent advances in deep learning and optical fabrication enable a novel end-to-end system design strategy, where optics and algorithms are jointly optimized by a deep neural network (36, 37, 49, 54). This AI-driven optimization at the system development stage unlocks new possibilities to enhance imaging performance. However, only a limited number of computational imaging systems have successfully demonstrated the ability to resolve cellular structures in dense tissue, and even fewer have achieved in vivo imaging capability.

**Fig. 1.**
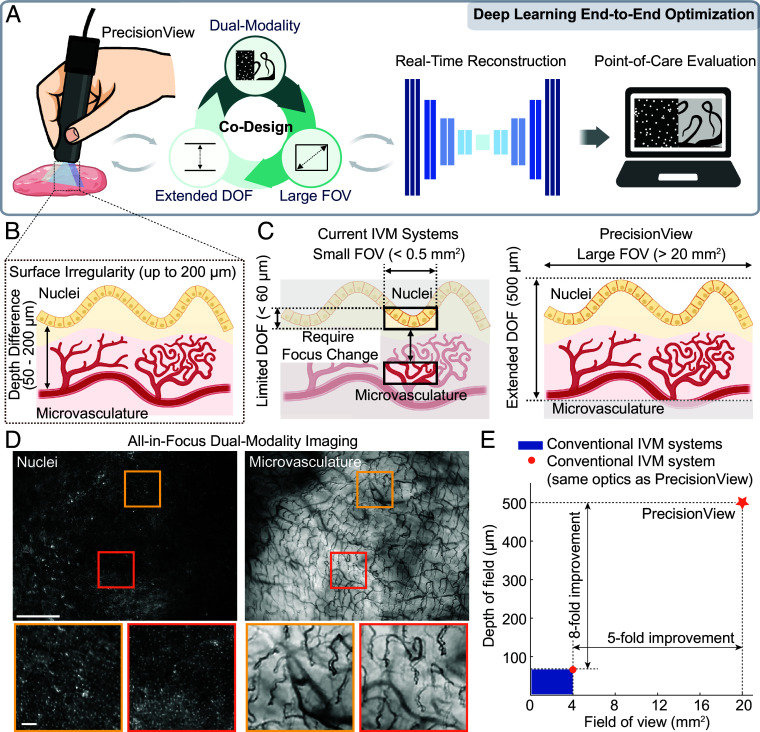
PrecisionView is a compact AI-powered handheld endomicroscope for real-time in vivo neoplasia detection at the point of care. (*A*) Schematic of the design principle and the imaging setup. PrecisionView is a compact, handheld, and simple-to-use endomicroscope designed for high-resolution, noninvasive imaging of epithelial tissue. By leveraging a deep learning framework to co-optimize hardware and software, PrecisionView enables fluorescence imaging of cell nuclei and reflectance imaging of microvasculature, while achieving significantly increased FOV and DOF compared to conventional systems. The deep learning reconstruction algorithm provides real-time video-rate image reconstruction for immediate microscopic evaluation of suspicious lesions, aiding in precancer and cancer detection at the point of care. (*B*) In vivo imaging of epithelial cell nuclei and microvasculature requires accommodation of surface irregularities (axial variations of up to 200 µm) and a broad range of depth differences between epithelial nuclei and subsurface microvasculature (typically 50 to 200 µm). (*C*) For existing conventional IVM systems, the FOV is fundamentally restricted which makes it challenging to assess large, heterogeneous lesions at risk. Due to their limited DOF, conventional high-resolution IVM systems require adjustment of the focal position to visualize cell nuclei and microvasculature at different depths. By integrating deep learning end-to-end optimization and simple off-the-shelf optics, PrecisionView overcomes these limitations and achieves an extended FOV and DOF. This enables rapid, coregistered mapping of cell nuclei and microvasculature in a large tissue area at cellular resolution. (*D*) Representative PrecisionView image of oral mucosa from a healthy volunteer acquired over a single 5.2 mm × 3.9 mm FOV. Nuclear morphology (fluorescence) and microvasculature (label-free reflectance) are alternatively visualized within the same coregistered FOV. The system provides a 500 µm DOF with 4 µm resolution, enabling all-in-focus imaging across uneven tissue surfaces. Boxed regions correspond to the zoomed panels. [Scale bars, full image, 1 mm; zoom-in views, 100 µm.] (*E*) Comparison of FOV and DOF between PrecisionView and current IVM systems. By incorporating deep learning end-to-end optimization, PrecisionView achieves a fivefold increase in FOV and an eightfold improvement in DOF compared to current IVM systems with similar resolution. Without the phase mask and image reconstruction, a conventional endomicroscope using the same conventional optics demonstrates a 4 mm^2^ effective FOV and 80 µm DOF.

In this study, we build upon these concepts and extend them toward a clinically relevant imaging platform by integrating end-to-end optical–computational optimization with a miniaturized optical system suitable for handheld operation. We present PrecisionView, a compact, AI-powered handheld endomicroscope designed to address the fundamental limitations of conventional IVM and improve in vivo early cancer detection at the point of care. By leveraging a deep learning end-to-end optimization framework, we simultaneously optimized both the phase mask design and reconstruction algorithm using a deep neural network. This framework is specifically optimized to incorporate dual-modality fluorescence and reflectance imaging, enabling complementary visualization of nuclear morphology and microvasculature with substantially increased FOV and DOF (Fig. 1 and *SI Appendix*, Fig. S1). The wavefront-encoding phase mask is positioned at the Fourier plane of the system to provide phase modulation, generating spatially- and depth-invariant point spread functions (PSFs). The resulting image degradation is then corrected by the reconstruction algorithm, which performs image deconvolution and deblurring to restore fine image details. The reconstruction framework explicitly accounts for spatially varying PSFs across the FOV and employs an optimized neural reconstruction architecture, enabling robust imaging performance over a large FOV. By integrating deep learning end-to-end optimization and simple off-the-shelf optics, PrecisionView achieves a 5.2 mm × 3.9 mm FOV and a 500 µm DOF with 4 µm resolution, while maintaining a compact form factor and a $3,000 cost of goods. This represents approximately a fivefold increase in FOV and eightfold improvement in DOF compared to conventional IVM systems of similar resolution (Fig. 1*E*). With the 40-fold larger volumetric FOV and the capability of fluorescence and reflectance imaging, PrecisionView enables large-scale, high-resolution, coregistered mapping of cell nuclei and microvasculature without requiring refocusing (Fig. 1 *C* and *D*). In addition, we deployed the deep learning reconstruction algorithm to enable real-time all-in-focus visualization, which allows immediate microscopic evaluation of suspicious lesions at 15 frames per second (FPS), providing diagnostic guidance at the point of care (Movie S1). We experimentally validated performance of PrecisionView by imaging standard test targets, ex vivo porcine tongue specimens, and postmortem human breast specimens, demonstrating superior resolution across an extended FOV and DOF compared to a conventional system. Furthermore, we established the in vivo imaging capability of PrecisionView by imaging the oral cavity of healthy volunteers. Through handheld scanning and image stitching, we successfully mapped cellular and vascular features over a tissue area >1.3 cm^2^ at high resolution, revealing distinct features of human lip mucosa and tongue. To assess PrecisionView’s clinical applicability, we performed ex vivo imaging of freshly resected cervical specimens with precancerous lesions, generating maps covering tissue areas >2.8 cm^2^ via handheld scanning. The system clearly delineated microscopic features corresponding to anatomical structures and pathological abnormalities. These results highlight PrecisionView as a promising noninvasive, rapid diagnostic tool with potential for identification of early epithelial neoplasia at the point of care.

## Results

### PrecisionView System Design and End-To-End Optimization Architecture.

**Fig. 2.**
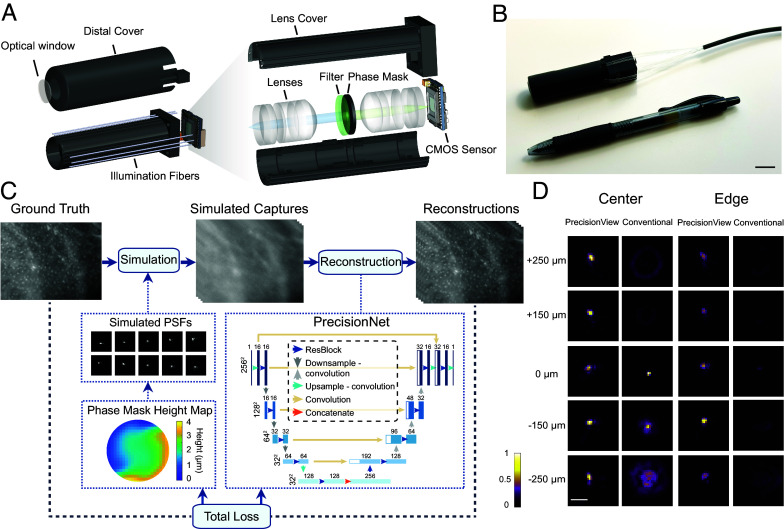
Deep learning end-to-end design of PrecisionView. (*A*) CAD rendering of PrecisionView. The device incorporates a simple conjugated microscope design with off-the-shelf lenses, a bandpass filter, an end-to-end optimized phase mask, and a miniature complementary metal-oxide semiconductor (CMOS) sensor. The phase mask is positioned at the Fourier plane of the system to enable phase modulation. Fourteen illumination fibers, attached externally to the lens cover, deliver light from a dual-color LED light source. A protective system cover with a sapphire optical window ensures safe contact imaging while maintaining the optimal working distance. (*B*) Photo of the assembled PrecisionView prototype. (Scale bar, 10 mm.) (*C*) End-to-end deep learning framework for joint optimization of the phase mask and reconstruction algorithm. At the optical layer, the end-to-end network simulates the PSFs encoded by the phase mask, generating simulated image captures within the targeted DOF. These simulated captures are then processed by the sequential reconstruction network (PrecisionNet), producing in-focus images. The end-to-end network simultaneously optimizes both the phase mask design and the reconstruction algorithm by minimizing the loss between the network output and ground truth. (*D*) Experimentally captured PSFs of PrecisionView compared to a conventional system without the phase mask, across the 500 μm depth range at the center and edge (2.5 mm from the center) of the FOV. The trained phase mask enables PrecisionView to generate spatially- and depth-invariant PSFs, facilitating the network digital layer to perform image reconstruction and extend the FOV and DOF. (Scale bar, 20 μm.)

PrecisionView enables direct, in-focus imaging of different morphological features over a large FOV (5.2 mm × 3.9 mm) and extended DOF (500 µm), utilizing a compact optical layout with end-to-end co-optimization of a phase mask and reconstruction algorithm. The system employs off-the-shelf achromatic lenses and a conjugated optics design, with identical optical designs used for both the microscope objective and tube lens (55). These components are aligned and housed within a custom 3D-printed enclosure (Fig. 2*A*). Each of the objective and tube lens assemblies consists of three 12 mm diameter achromatic lenses (*SI Appendix*, Fig. S2). A 520/40 nm bandpass emission filter and a jointly optimized phase mask are placed between the objective and tube lens to form the imaging path.

System illumination is provided by 14 optical fibers symmetrically positioned around the lens housing. The fibers are bundled together and connected to a light source containing blue and green light-emitting diodes (LEDs), The two LEDs are paired with appropriate filters and combined via a dichroic mirror to enable rapid switching between blue and green illumination for fluorescence and reflectance imaging, respectively (*SI Appendix*, Fig. S3). The optical fibers are arranged to ensure uniform illumination across the entire FOV (*SI Appendix*, Fig. S4). The entire system is enclosed in a distal cover with a front-facing sapphire window that allows direct contact with tissue surfaces for imaging. This cover can be readily removed for disinfection (Fig. 2*A*). The fully integrated system has a compact form factor with 14 mm diameter and 7 cm length, comparable to the size of a pen (Fig. 2*B*).

The phase mask design and image reconstruction algorithm were jointly modeled and optimized using an end-to-end deep learning framework (Fig. 2*C* and *SI Appendix*, Fig. S5). The first layer of the network implements a physics-informed model that simulates the image formation process of a conventional microscope with a learnable phase mask at the Fourier plane (*SI Appendix*). This simulation accounts for both fluorescence and reflectance imaging modes, and models defocus across 21 discrete depths spanning the 500 µm DOF. Following the optical simulation layer, digital reconstruction is performed by a modified U-Net architecture, which we refer to as PrecisionNet. This network incorporates several enhancements compared to basic U-Net architecture, including residual blocks, a pyramid pooling module (PPM), and pixel-shuffle convolution layers to improve feature representation and image quality (*SI Appendix*). The training dataset used for end-to-end optimization includes microscopy images of a wide range of features from both fluorescence and reflectance imaging modalities to optimize dual-modality imaging with PrecisionView (*SI Appendix*). The phase mask height profile and the reconstruction algorithm were simultaneously optimized during end-to-end training to minimize the loss.

Following end-to-end training, the optimized phase mask was fabricated using two-photon photolithography (*SI Appendix*, Fig. S6) and integrated into the PrecisionView system (*Materials and Methods* and *SI Appendix*). Once the system was fully assembled, a one-time calibration was performed by capturing PSFs at different depths and spatial locations across the FOV (Fig. 2*D*). These experimentally measured PSFs were then used to fine-tune PrecisionNet for improved reconstruction accuracy (*Materials and Methods*). Compared to a conventional system with identical optics but without the trained phase mask, the captured PSFs of PrecisionView exhibited significantly improved contrast and consistency across the 500 µm DOF, both at the center and edges of the FOV. The modulated PSFs facilitate the network digital layer to perform image reconstruction and extend the FOV and DOF of the system (*SI Appendix*, Figs. S7 and S8).

### PrecisionView System Characterization.

**Fig. 3.**
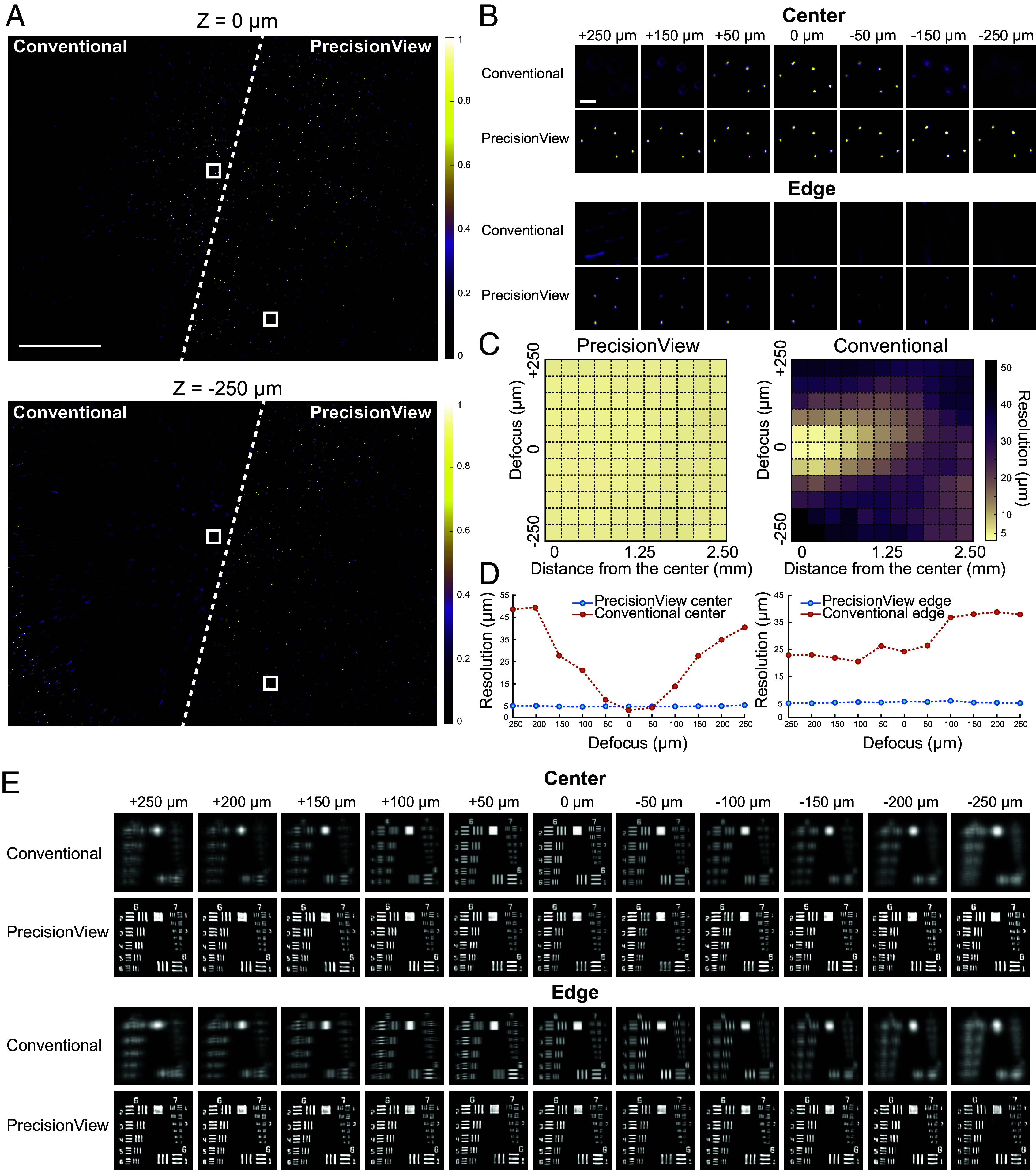
Characterization of the imaging performance of PrecisionView compared to a conventional system. (*A*) Experimentally captured images of 4 μm diameter fluorescent beads using PrecisionView and the conventional system at imaging depths of 0 μm and to 250 μm. (Scale bar, 1 mm.) (*B*) Zoom-ins of the boxed regions in panel a across a 500 μm depth range. PrecisionView maintains high-resolution visualization of beads consistently across this depth range, both at the center and edge of the FOV. (Scale bar, 50 μm.) (*C*) Experimentally measured lateral resolution across the entire DOF and FOV of PrecisionView and the conventional system. PrecisionView achieves a consistent lateral resolution of ~4 μm, significantly outperforming the conventional system. (*D*) Comparison of the lateral resolution of PrecisionView and the conventional system across a 500 μm depth range, evaluated at the center (*Left* plot) and edge (*Right* plot) of FOV. (*E*) Experimentally captured images of a USAF test target using PrecisionView and the conventional system across a 500 μm depth range at the center and edge of the FOV. PrecisionView consistently resolves group 7, element 1 (3.91 μm line width).

**Fig. 4.**
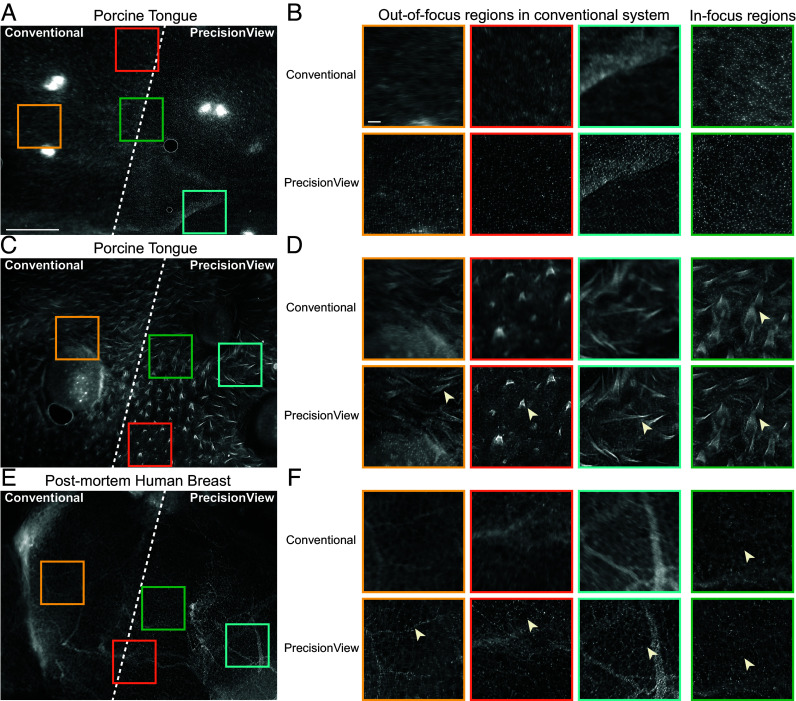
Representative ex vivo images of fresh porcine tongue and human breast specimens acquired with PrecisionView and the conventional system. (*A*) Representative images of a porcine tongue specimen. (Scale bar, 1 mm.) (*B*) Zoom-ins of annotated ROIs in panel A. (Scale bar, 100 μm.) (*C*) Representative images of a different porcine tongue specimen showing distinct structural features. (*D*) Zoom-ins of annotated ROIs in panel C. Arrows indicate representative papillary features visualized within the selected ROIs. (*E*) Representative images of a postmortem human breast specimen. (*F*) Zoom-ins of annotated ROIs in panel E. Arrows indicate representative adipocytes visualized within the selected ROIs. Results demonstrate that PrecisionView consistently resolves distinct cellular and epithelial morphology with high resolution across a large FOV in various tissue types, while the same features are blurred in various regions of FOV in images acquired with the conventional system due to geometric aberrations or limited DOF. In tissue regions that are in focus in the conventional system (green ROIs), the same cellular features are clearly resolved when imaged with PrecisionView.

We characterized the performance of PrecisionView using standardized test targets and fluorescent bead samples and found that the system achieves a lateral resolution of approximately 4 µm across a 500 µm DOF, with uniform performance maintained throughout the entire FOV. To ensure a fair comparison, we fabricated a reference system, referred to as Conventional in Figs. 2–4, using the same optical, illumination, and housing components as PrecisionView, but without the phase mask or the reconstruction algorithm.

We first imaged a glass slide with 4 µm diameter fluorescent beads spread across the slide using both PrecisionView and the conventional system (Fig. 3*A*). At the focal plane, both systems produced sharp, in-focus images at the center of the FOV. However, the conventional system showed noticeable image degradation toward the edges of the FOV, while PrecisionView maintained consistent image quality across the entire FOV. When imaging at defocused depths, only PrecisionView was able to recover clear images at both central and peripheral regions (Fig. 3 *A* and *B*), highlighting its superior depth invariance and reduced sensitivity to optical aberrations.

We quantitatively evaluated the lateral resolution of PrecisionView and compared it to the conventional system across the full FOV and DOF. We imaged a glass slide with 1 µm diameter fluorescent beads spread across the slide, and the full width at half maximum (FWHM) of the bead intensity profiles was calculated at various spatial and depth positions for both systems (*Materials and Methods*). PrecisionView maintained a consistent lateral resolution of approximately 4 µm throughout the 500 µm depth range and across the entire 20 mm^2^ FOV, demonstrating its robust performance in both spatial and axial dimensions (Fig. 3 *C* and *D*). In contrast, the conventional system exhibited high resolution only near the center of the FOV due to geometric aberrations and within a limited axial range of approximately 60 µm. Beyond this range, the lateral resolution of the conventional system degraded rapidly. These results highlight PrecisionView’s superior ability to maintain high-resolution imaging over approximately a fivefold expanded FOV and eightfold extended DOF, enabled by the optimized phase mask and computational reconstruction.

We further validated the performance of PrecisionView by imaging a negative 1951 USAF resolution target and comparing results to those from the conventional system (Fig. 3*E*). As expected, the conventional system was able to resolve the target elements at the focal plane; however, significant defocus blur appeared as the target was translated axially away from the focal plane, and image quality degraded toward the edges of the FOV. In contrast, PrecisionView consistently resolved Group 7, Element 1 (3.91 µm line width) across the entire 500 µm DOF, both at the center and edges of the FOV. These results further confirm the ability of PrecisionView to maintain high-resolution, in-focus imaging over an extended imaging volume.

### Ex Vivo Imaging of Porcine Tongue and Human Breast Specimens.

PrecisionView consistently resolves cellular features in freshly excised tissue specimens with high resolution across a large FOV. When imaging the same tissue regions, PrecisionView outperformed the conventional system configuration, which had no end-to-end optimized phase mask and reconstruction algorithm (Fig. 4).

For these experiments, fresh porcine tongue and postmortem human breast tissues were cut with a scalpel and topically stained with 0.01% (w/v) proflavine solution in phosphate-buffered saline (PBS) using cotton-tipped applicators. The specimens were then placed on a glass window, and the system without distal cover was used to image from below to visualize the stained tissue surface through the glass window. For comparison, the same tissue regions were imaged without refocusing using the PrecisionView system and the conventional system configured with an identical optical layout, but without the phase mask. Fluorescence imaging was enabled using a blue LED light source for both systems.

Representative images from both tissue types are shown in Fig. 4, with three regions of interest (ROIs) selected per image for zoom-in visualization. We stained and imaged the squamous epithelium of the porcine tongue using both systems. Images of the squamous epithelium of porcine tissue acquired with PrecisionView clearly reveal cell nuclei with consistently high resolution across the entire FOV, while images acquired with the conventional system show blurred cellular features in portions of the FOV. (Fig. 4 *A* and *B*). In regions of the porcine tongue with more complex features, PrecisionView effectively resolves multiple features including cell nuclei, keratinized epithelium, and papillae (Fig. 4 *C* and *D*). In postmortem human breast tissue, we imaged the cut surface of the sliced specimen. PrecisionView distinctly visualizes adipose tissue and cell nuclei, which appear poorly resolved in various regions of images acquired with the conventional system (Fig. 4 *E* and *F*). In tissue regions that are in focus in the conventional system (green ROIs), PrecisionView accurately reveals the same cellular structures. These results demonstrate that PrecisionView enables in-focus, high-resolution imaging over a large FOV in fresh tissue specimens. Images acquired with PrecisionView consistently reveal epithelial and nuclear morphology across diverse tissue types. In contrast, images acquired with the conventional system show significantly blurred structures in many regions within the FOV due to geometric aberrations toward the periphery and the limited DOF. These effects reduce the ability to resolve nuclear boundaries and other fine morphological details in conventional images, particularly when imaging uneven tissue surfaces.

### In Vivo Imaging of the Oral Cavity in Healthy Volunteers.

**Fig. 5.**
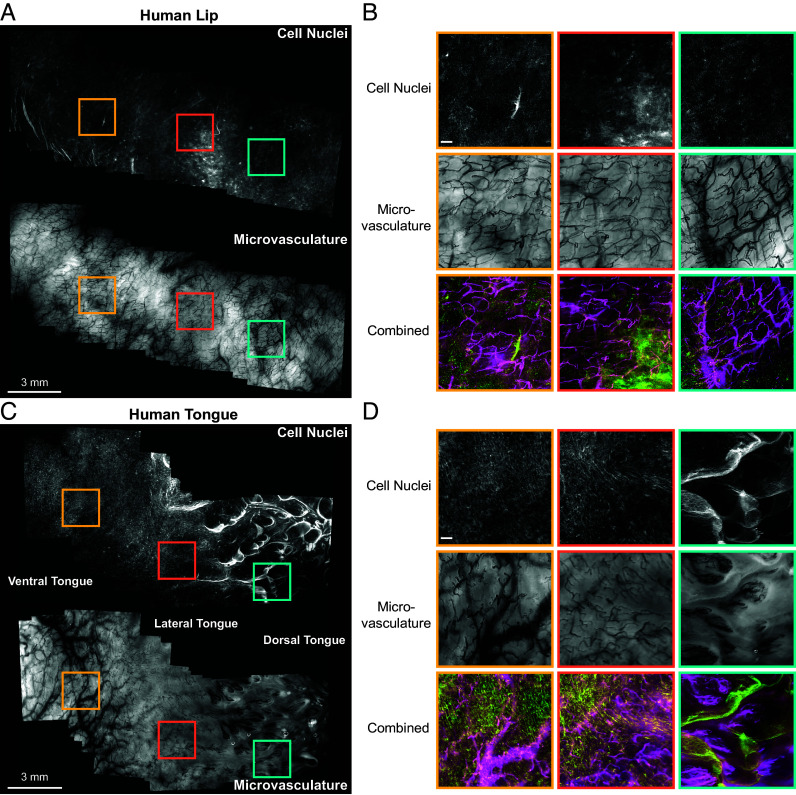
In vivo large-scale, high-resolution, coregistered mapping of cell nuclei and microvasculature in the oral cavity of healthy volunteers using handheld PrecisionView imaging. (*A*) Stitched maps of cell nuclei and microvasculature of the lip mucosa of a healthy volunteer, covering a tissue area of ~1.3 cm^2^. Video acquisition time, ~1 min. (Scale bar, 3 mm.) (*B*) Zoom-ins of annotated ROIs in panel *A*, showing separate and combined high-resolution images of cell nuclei and microvasculature for each ROI. (Scale bar, 200 μm.) (*C*) Stitched maps of cell nuclei and microvasculature of the tongue of a healthy volunteer, covering a tissue area of ~1.3 cm^2^. Video acquisition time: ~1 min. (Scale bar, 3 mm.) (*D*) Zoom-ins of annotated ROIs in panel *C*, showing separate and combined high-resolution images of cell nuclei and microvasculature for each ROI. PrecisionView enables clear visualization of distinct tissue structures across the ventral, lateral, and dorsal tongue. (Scale bar, 200 μm.)

We validated the in vivo imaging performance of PrecisionView in the oral cavity of healthy volunteers by visualizing cell nuclei and microvasculature in real time. The system was placed in gentle contact with the oral mucosa and scanned manually across large tissue areas while performing dual-modality imaging. With the large DOF, coregistered high-resolution images of cell nuclei and microvasculature across a large tissue area could be acquired without refocusing. As the system scanned the mucosal surface, the large FOV of each frame and the high video acquisition rate (~15 FPS) provided substantial frame-to-frame overlap, enabling seamless stitching of dual-modality images to generate large-scale, coregistered maps (>1.3 cm^2^) of the oral mucosa (Fig. 5). These maps clearly reveal distinct cellular and vascular features associated with anatomic variations with high spatial resolution.

Before imaging, tissue regions of interest in the oral cavity were topically stained with 0.01% (w/v) proflavine in PBS using cotton-tipped applicators. While imaging, we placed the PrecisionView system in gentle contact with the oral mucosa and manually scanned the stained tissue regions. The blue LED and green LED were alternated at 1 Hz to acquire fluorescence images of cell nuclei and reflectance images of microvasculature during scanning. Leveraging the system’s large FOV and extended DOF, coregistered high-resolution images were acquired without refocusing and stitched to generate coregistered maps of cell nuclei and microvasculature (Movie S2). Microvascular blood flow is clearly observed in several vessels as shown in the video. Representative stitched images of the lip mucosa covering a tissue area of ~1.3 cm^2^ are shown in Fig. 5*A*. In the zoomed-in views of annotated ROIs (Fig. 5*B*), coregistered nuclear and microvascular structures are clearly visualized at high resolution. The extended DOF allows simultaneous acquisition of in-focus images of both cell nuclei and microvasculature. The large-scale maps reveal localized variations in the architecture of the microvasculature. The microvasculature map acquired from regions around the yellow and orange ROIs in the lip predominantly reveal capillary loops, while the microvasculature maps acquired from the blue ROI show a higher density of branching vessels.

Scanning across the ventral, lateral, and dorsal tongue epithelium with the PrecisionView system further demonstrated its ability to reveal distinct, localized tissue features at high resolution (Fig. 5 *C* and *D* and Movies S3 and S4). On the ventral tongue (yellow ROI), both capillary loops and larger vessels are visualized with clearly resolved cell nuclei. The lateral tongue (orange ROI) shows similar features of cell nuclei and smaller vessels compared to the ventral tongue. While fluorescence imaging alone is not able to reliably distinguish between ventral and lateral tongue regions, the additional contrast from reflectance imaging significantly enhances the ability to differentiate these anatomic locations, highlighting the potential of dual-modality imaging. In images from the dorsal tongue (blue ROI), PrecisionView shows keratinized epithelium in fluorescence images and capillary loops embedded within the keratinized tissue in reflectance mode. Imaging of the ventral tongue surface, which is less keratinized and exhibits a smoother epithelial morphology than the dorsal surface, reveals more clearly resolved nuclear patterns (Movie S4). In contrast, the dorsal tongue surface is more corrugated and heavily keratinized, which can introduce additional scattering and local aberrations that reduce the apparent clarity of nuclear features in some regions. The combination of coregistered fluorescence and reflectance images clearly reveal the microscopic structure of papillae in the dorsal tongue.

### Ex Vivo Imaging of Cervical Specimens With Precancerous Lesions.

**Fig. 6.**
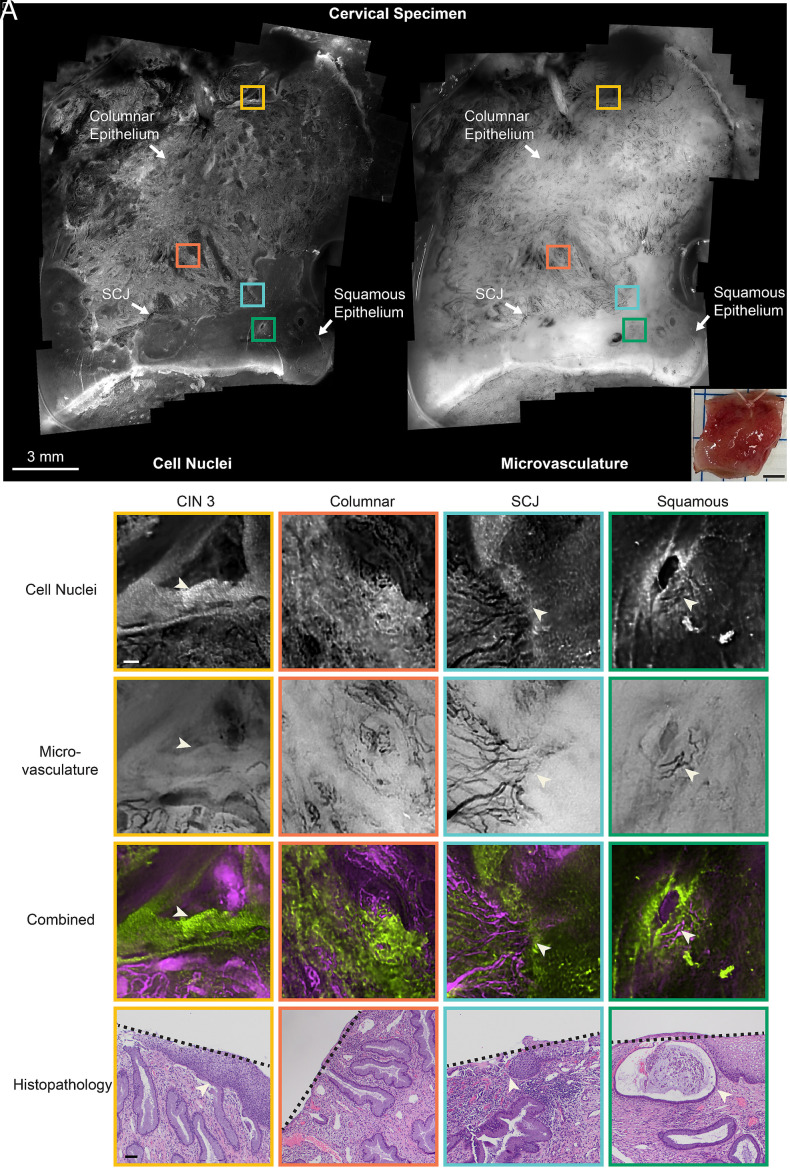
Ex vivo coregistered maps of cell nuclei and microvasculature from a freshly resected cervical specimen with precancerous lesions using PrecisionView. (*A*) Stitched dual-modality maps of cell nuclei and microvasculature from the epithelial surface of a freshly resected cervical specimen, covering a tissue area of ~2.8 cm^2^. The coregistered maps delineate the columnar epithelium, the squamocolumnar junction (SCJ), and squamous epithelium. A photograph of the imaged specimen is shown at the *Bottom Right*. Video acquisition time, ~5 min. Scale bar of stitched maps, 3 mm. Scale bar of specimen photograph, 5 mm. (*B*) Zoom-ins of annotated ROIs in panel *A*, showing separate and combined high-resolution images of cell nuclei and microvasculature for each ROI. PrecisionView reveals a focal high-grade squamous intraepithelial lesion (CIN 3) in the background of columnar epithelium (indicated by arrows). The system also distinguishes columnar and squamous epithelium and accurately identifies the SCJ (indicated by arrows). An endocervical gland can also be observed within the green ROI located in the normal squamous region (indicated by arrows). Corresponding histopathology from the same tissue regions confirmed the findings from PrecisionView. PrecisionView images are acquired parallel to the tissue surface, whereas histologic sections are cut perpendicular to the surface. Black dashed lines in the pathology images indicate the imaging surface to illustrate this orientation difference. (Scale bars, 100 μm.)

To validate the capability of PrecisionView to detect precancerous lesions, we performed imaging on freshly resected human cervical specimens with squamous intraepithelial lesions (precancer). Following manual scanning of whole specimens and image stitching, dual-modality maps of the epithelium of the entire cervical specimen were generated for high-resolution assessment of epithelial and vascular structures (Fig. 6 and Movie S4). Coregistered maps of the cell nuclei and microvasculature clearly delineated key anatomic structures, including columnar and squamous epithelium, as well as the squamocolumnar junction (SCJ). High-grade squamous intraepithelial lesions were clearly distinguishable from surrounding benign tissue, demonstrating the clinical potential of PrecisionView for noninvasive detection of neoplasia.

In this pilot study, cervical specimens were obtained via loop electrosurgical excision procedure (LEEP) or cold knife cone (CKC) biopsy performed for the treatment of cervical dysplasia (precancer). PrecisionView imaging was carried out immediately after excision. Prior to imaging, the epithelial surface of ectocervical specimens was first cleaned with sterile swabs and saline to remove residual debris, then topically stained with 0.01% (w/v) proflavine solution in PBS. During imaging, the handheld PrecisionView system was placed in gentle contact with the stained tissue surface and scanned manually across the entire specimen. The blue LED and green LED were alternated at 1 Hz to enable dual-modality imaging during tissue scanning. Acquired images were stitched to produce high-resolution, coregistered maps of nuclear and microvascular features. Representative stitched images of a cervical specimen are shown in Fig. 6. Additional results are shown in *SI Appendix*, Fig. S8. Similar to the in vivo oral imaging, PrecisionView enabled simultaneous high-resolution visualization of both cell nuclei and microvasculature. The large-scale dual-modality maps and corresponding zoom-ins clearly delineate the columnar and squamous epithelium and highlight the SCJ. As shown in Fig. 6*B*, glandular structures and dense stromal vasculature are clearly visualized in the orange ROI within the columnar epithelium. The combination of nuclear and vascular features reveals the architecture of endocervical glands. In contrast, images of the normal squamous epithelium show uniform stratified cell nuclei and sparse capillary loops. An endocervical gland can also be observed within the green ROI located in the squamous region (indicated by arrows in Fig. 6*B*). The SCJ is distinctly visualized as the transition boundary between columnar and squamous epithelium (indicated by arrows in Fig. 6*B*). Imaging findings were confirmed by histopathological analysis of the same tissue regions. The extended DOF of PrecisionView enabled consistent, in-focus imaging across the entire tissue surface without the need for refocusing, despite significant variations in the microvasculature depth across different anatomic sites (20). Notably, an island of suspicious squamous mucosa located within the columnar tissue region was highlighted in the dual-modality maps and corresponding zoom-ins (indicated by arrows in Fig. 6*B*). The suspicious squamous tissue showed dense nuclei, enhanced fluorescence, and increased vascular density compared to normal squamous tissue. Histopathological analysis confirmed the region as a high-grade squamous intraepithelial lesion (CIN 3). The correspondence between PrecisionView images and histology is influenced by differences in imaging orientation. PrecisionView visualizes tissue parallel to the tissue surface, whereas histologic sections are obtained perpendicular to the surface. Despite this difference, similar architectural features can be identified in Fig. 6. These results demonstrate the potential of PrecisionView for noninvasive, high-resolution imaging of large tissue epithelial surfaces, supporting its applications as a point-of-care diagnostic tool for detecting precancer and early cancer.

## Discussion

In this study, we present a compact, affordable ($3,000), AI-powered endomicroscope capable of high-resolution, dual-modality imaging of cell nuclei and microvasculature with a large FOV (>20 mm^2^) and extended DOF (500 µm) for in vivo precancer and cancer detection. The present work builds upon prior phase-mask–based extended DOF approaches while extending these concepts toward a clinically deployable in vivo imaging system. In contrast to earlier implementations that primarily focused on single-modality imaging and spatially invariant optical models, the proposed framework incorporates dual-modality imaging, accounts for spatially varying PSFs across a large FOV, and integrates these elements within a miniaturized optical platform suitable for handheld operation. The reconstruction algorithm was also updated with the PrecisionNet architecture. Importantly, the deployment of real-time deep learning reconstruction enables immediate all-in-focus visualization of nuclear and vascular features, facilitating point-of-care evaluation of epithelial lesions. These developments highlight how optical–computational imaging approaches can be translated from proof-of-concept demonstrations to clinically relevant imaging systems.

Conventional IVM systems face fundamental trade-offs between resolution, FOV, DOF, and system form factor. The extended FOV demonstrated by PrecisionView reflects an increase in the effective usable field rather than simply an expansion of the geometric field defined by the optics and sensor. In compact optical systems, off-axis aberrations typically degrade image quality toward the periphery, reducing the usable FOV. PrecisionView mitigates field-dependent aberrations and maintains more uniform resolution across the sensor area, thereby enlarging the effective imaging region. The ultimate geometric FOV, however, remains determined by the optical design, numerical aperture, and sensor size. Other methods have been used to extend the DOF by enabling axial scanning using an electrically tunable lens (56–58) or a deformable mirror (59). However, these approaches increase system complexity and cost and are difficult to implement in handheld imaging systems due to their sensitivity to motion. In contrast, PrecisionView achieves significant improvement in both FOV and DOF through a simple optical layout and compact design, which are otherwise unachievable with conventional optics. Although PrecisionView is designed to provide extended DOF, its peak in-focus contrast at the exact focal plane may be modestly lower than that of a conventional diffraction-limited system without phase encoding. This behavior reflects the trade-off of wavefront coding approaches, in which optical energy is redistributed to achieve depth invariance at the cost of reduced peak sharpness at the nominal focal plane. There is a tradeoff between optical sharpness at the nominal focal plane and improved preservation of spatial frequency information across defocus. In controlled resolution-target measurements performed precisely at focus, the conventional system can therefore exhibit slightly higher apparent sharpness. However, in biologically relevant imaging conditions, where tissue surfaces are irregular and local defocus is common, the extended DOF of PrecisionView maintains more consistent effective resolution across depth variations. Thus, the system is optimized for robust performance over a wide axial range rather than maximizing contrast at a single focal plane. PrecisionView currently is designed primarily for superficial epithelial imaging where scattering-induced aberrations remain moderate. In deeper or highly heterogeneous tissue, scattering can introduce additional distortions that alter the system PSF. Future work could improve robustness by incorporating simulated or experimentally measured scattering conditions into the training process, enabling the reconstruction model to better generalize across tissues with diverse optical properties. The PrecisionView system can be readily assembled using off-the-shelf components and a customized phase mask at low cost. Extended DOF is achieved with each image capture, facilitating handheld clinical use.

Unlike most computational microscopy techniques that rely on extensive postprocessing, PrecisionView integrates rapid deep learning reconstruction directly into the video acquisition pipeline. Although the camera can support video acquisition rates of up to 30 FPS, the effective acquisition frame rate is primarily limited by the long exposure time (~65 ms) required for fluorescence imaging, corresponding to a frame rate of approximately 15 FPS in most experiments when performing dual-modality imaging. No postsensor electronic gain was applied during acquisition, as the fluorescence signal from proflavine-stained tissue provided sufficient photon flux under these conditions. Avoiding electronic gain minimized amplification of read and fixed-pattern noise. With an exposure time of ~65 ms, motion blur is visible in the videos when the probe is translated across tissue (Movies S1–S5). To mitigate this effect, we implemented a handheld stop-and-go acquisition strategy in which the system is held stationary at each imaging site for ~2 s before translating it to the next location. This enables the capture of sharp, motion-free images at each site. When operating only in reflectance mode for imaging microvasculature, PrecisionView can readily achieve faster video acquisition at 30 FPS with a substantially shorter exposure time and reduced motion blur. The real-time reconstruction speed is determined by the computation capability of the hardware. The PrecisionNet achieves real-time reconstruction at 22 FPS on a workstation with an RTX 4090 GPU, and at 7 FPS on a laptop with an RTX 3080 GPU. The customized GUI allows raw image acquisition at the maximum frame rate while displaying reconstructed frames at a lower rate. The reconstruction frame rate only affects the visualization rate of reconstructed images but not the degree of motion blur, which is determined solely by the exposure time during acquisition. The current exposure time is primarily constrained by the available excitation power for fluorescence imaging and the resulting signal intensity. In particular, excitation efficiency is limited by the transmission losses through the illumination fibers. In future prototypes, we plan to integrate miniature LEDs at the distal end of the PrecisionView. This design would provide orders-of-magnitude higher excitation power at the tissue surface while remaining within established safety limits. The resulting increase in fluorescence signal intensity could allow substantially shorter exposure times, thereby reducing motion blur, improving signal-to-noise ratio, and facilitating more accurate coregistration of dual-modality images.

Although image mosaicking has been used to expand the FOV of conventional IVM systems, the total stitched imaging area remains limited (<10 mm^2^) due to the small FOV of each individual image acquisition and challenges in maintaining continuous scanning without abrupt positional shifts (21, 34, 35). In contrast, the FOV and extended DOF of PrecisionView simplify large-area image mosaicking during manual scanning. In conventional IVM systems, the small FOV often requires tightly controlled probe motion and small frame-to-frame shifts to maintain sufficient overlap for reliable stitching. Previous studies have reported frame shifts on the order of hundreds of micrometers to ensure accurate registration (21). Our experiments indicate that an interframe overlap of approximately 30% is sufficient for robust mosaicking with PrecisionView, corresponding to frame shifts of ~3 to 3.5 mm given the 5.2 mm × 3.9 mm FOV. The larger imaging area and reduced sensitivity to axial defocus preserve recognizable structural landmarks across frames, thereby relaxing scanning constraints and facilitating practical large-area imaging in clinical settings. Moreover, the dual-modality capability of PrecisionView allows precise, coregistered detection of abnormal changes of both cell nuclei and microvasculature across large tissue surfaces to enhance clinical performance. To generate coregistered maps, we employed a stop-and-go acquisition pattern to scan the tissue (Movies S1–S4) which minimized the motion artifacts and ensured the acquisition of coregistered images of cell nuclei and microvasculature at each tissue site. Alternating between the two imaging modes at 1 Hz facilitates the real-time visualization and assessment of both nuclear and vascular features. Because fluorescence and reflectance signals are acquired sequentially, rapid probe motion may introduce minor interframe misregistration and perceptual switching between modalities can be visually distracting during continuous viewing. The current implementation mitigates motion artifacts using a handheld stop-and-go acquisition strategy and supports single-mode operation upon clinician request. Future systems could further improve robustness and usability through tighter hardware synchronization between image frame acquisition and dual-color illumination, increased LED switching frequency for smoother visualization, user-controlled switching modes, or simultaneous dual-channel detection. With large-scale coregistered dual-modality maps, PrecisionView reveals detailed tissue architecture with high resolution, such as tongue papillae and endocervical glands. PrecisionView also accurately delineates various tissue anatomical subregions, which is critical for improving cancer detection. For example, since most squamous cell carcinomas of the cervix arise in the transformation zone (60), the ability of PrecisionView to clearly outline the SCJ enables more accurate assessment of high-risk regions and potential for improved identification of precancerous lesions.

With the ability for multiscale dual-modality evaluation of tissue, PrecisionView is designed to highlight specific microstructural hallmarks of epithelial neoplasia, including nuclear morphology and vascular architecture. The pathological information provided by PrecisionView has the potential to facilitate accurate diagnosis without the need for conventional biopsy. In practice, PrecisionView is intended to complement rather than replace standard endoscopic imaging. Integrating white-light illumination or multimodal image fusion would provide familiar anatomical context while preserving enhanced microstructural contrast, potentially improving clinical interpretability and diagnostic confidence. By combining macroscopic surface assessment with cellular-level detail, a multimodal approach could enable more informed targeting of suspicious regions and reduce reliance on subjective visual assessment alone. PrecisionView has the potential to facilitate patient triage by determining which patient with a positive screening test needs a biopsy, provide highly precise guidance for biopsy location, and enable accurate surgical margin assessment at the point of care. It could help clinicians and surgeons to make immediate decisions such as whether additional resection is needed for positive margins, potentially reducing the need for on-site pathology or frozen section which is not available in many clinics and hospitals globally.

The practical deployment of computational imaging systems requires robustness to routine handling and environmental variation. PrecisionView is enclosed within a rigid housing and incorporates a replaceable distal protective cap, allowing surface debris or minor scratches to be addressed without affecting internal alignment or calibration. Minor contamination within the optical path primarily introduces low-level scattering or attenuation and does not substantially alter the phase-mask encoding characteristics. During routine operation over more than one year at multiple locations, including clinical settings in the hospital, no recalibration or retraining was required, indicating stable reconstruction performance under diverse use conditions.

PrecisionView is designed to operate with minimal computational power. We used small-size images (368 × 480 pixels) for network training, allowing the model to be trained on standard workstations equipped with a GPU with 8 GB of memory. After one-time training, the trained network and customized user interface are deployed on a laptop, maintaining full-speed raw image acquisition and video-rate reconstruction. The frame rate of reconstruction is dependent on hardware specifications. PrecisionView has the potential to improve early detection for a variety of epithelial cancers, including cervical, oral, and anal cancer. The modular and versatile design of PrecisionView allows for broad imaging applications with minimal system modification. For example, the system can be adapted to use alternative contrast agents such as methylene blue, a dye with FDA approval for use in chromoendoscopy, to image cell nuclei with reflectance imaging (61). The largest contributor to the system size of our current prototype is the off-the-shelf CMOS sensor packaging (approximately 17 mm × 17 mm), while the sensor active area used for imaging is much smaller (5.2 mm × 3.9 mm). Future miniaturization could be achieved by adopting sensors with more compact packaging while maintaining a similar active area, together with smaller optical components. Our pilot study of ex vivo imaging in cervical specimens with precancerous lesions demonstrated PrecisionView’s ability to differentiate precancerous from benign tissue. Future in vivo clinical studies with larger sample sizes are warranted to rigorously assess the diagnostic performance of PrecisionView with statistical significance. With more clinical data, computer-aided diagnostic algorithms can be developed to automatically interpret PrecisionView images. This can further reduce the need for trained medical personnel, which is particularly valuable for improving cancer detection and early intervention in low-resource settings.

## Materials and Methods

### Optical Design and Device Fabrication.

The PrecisionView prototype was built utilizing a commercially available camera (XIMEA MU050MR-SY) with a monochrome Sony CMOS imaging sensor (IMX675 with 5 MP and 2.0 μm pixel size, readout noise of 4.0 e^−^, signal-to-noise ratio (SNR) of 40.2 dB, and sensitivity of 14,843 Digit/lx/s). The optical system of PrecisionView consists of two optical subassemblies, an emission filter (Chroma ET520/40 m), and a phase mask, all mounted in 3D-printed housings (Formlabs Form 3+, Black Resin). The objective subassembly (NA = 0.1) contains three achromatic lenses (#45-424, #49-658, #49-660, Edmund Optics), and the tube lens subassembly is identical to the objective lens and placed immediately after the emission filter and phase mask (Fig. 2*A* and *SI Appendix*, Fig. S2).

Illumination for the system was delivered via 14 plastic optical fibers (500 μm diameter, #02-532, Edmund Optics) arranged symmetrically around the imaging module and angled at 75 degrees to provide uniform illumination across the entire FOV (*SI Appendix*, Fig. S4). The measured excitation power at the designed working distance was approximately 0.15 mW/mm^2^ for blue light and 0.05 mW/mm^2^ for green light, resulting in a maximum illumination intensity at the skin of less than the threshold limit value specified by National Conference of Governmental Industrial Hygienists (ACGIH). All fibers are bundled and coupled to an LED-based illumination module comprising a blue LED (Mouser LZ4-40B208-0000) with an excitation filter (#84-705 Shortpass Filter, Edmund Optics), a green LED (Mouser LZ4-40G108-0000_G2) with a bandpass filter (Chroma ET520/40 m), and a dichroic mirror (Thorlabs DMLP490R, T_avg_ > 90%, R_avg_ > 95%) to enable switching between blue and green illumination (*SI Appendix*, Fig. S3). An Arduino (UNO R3) was used to synchronize the illumination. Fluorescence imaging was performed using the blue LED (center wavelength 457 nm) to match the excitation peak of proflavine. Reflectance imaging employed the green LED (center wavelength 523 nm) to enhance vascular contrast associated with hemoglobin absorption, and to remain compatible with the emission filter and the wavelength-dependent phase-mask design and calibrated forward model used for reconstruction. In our experiments, we observed no measurable background leakage in both fluorescence and reflectance imaging modalities. Tissue autofluorescence at the excitation wavelength was also very low and did not affect the imaging quality under the selected filters.

After assembly, the entire system was enclosed with a 3D-printed cap (Formlabs Form 3B, BioMed Black Resin), featuring a front-facing antireflection coated sapphire window (#20-633 Edmund Optics) for direct contact imaging. To maintain sterility and protect the device, the 3D-printed cap was disinfected following a standard high-level disinfection procedure using Cidex OPA (Advanced Sterilization Products) after each imaging session (62).

### End-To-End Training Framework.

A physics-informed deep neural network was developed to simultaneously optimize the phase mask design and the image reconstruction pipeline. This end-to-end framework includes a learnable optical layer followed by a reconstruction module, referred to as “PrecisionNet”, which performs the final image recovery. The phase mask height map and reconstruction algorithm were simultaneously optimized during end-to-end training to minimize the loss. Details of the learnable optical layer, PrecisionNet, the training dataset and implementation, and phase-mask fabrication are provided in the *SI Appendix*.

### Device Calibration and Network Fine-Tuning.

For each assembled system, a one-time calibration procedure was performed to record the experimental PSFs of the system prior to imaging, accounting for differences between simulated and experimental PSFs caused by alignment and fabrication tolerances. The point source used for calibration was a 3 μm pinhole array with 500 µm spacing that was illuminated by a green LED (Thorlabs, M530L4) placed behind an 80-degree holographic diffuser. The array was first brought into focus at the nominal focal plane and then scanned axially from −250 µm to +250 µm in 50 µm steps to generate focal stacks for training pair construction. At each axial position, 80 spatially distributed PSFs were captured simultaneously across the FOV. In total, 11 depths were calibrated. To improve signal-to-noise ratio, each calibration image was averaged over ten repeated acquisitions. The complete PSF calibration procedure required less than 30 min.

When the phase mask was incorporated into the system, we observed a significant reduction in spherical aberration. However, the PSFs continued to vary across the FOV, which degraded reconstruction performance when PrecisionNet was fine-tuned using only a single, centrally calibrated PSF. To address this issue, we utilized a comprehensive set of experimentally calibrated PSFs captured across different depths and spatial locations for network fine-tuning. During retraining, these experimentally measured PSFs were used in a forward model to generate simulated ground truth–capture image pairs. PrecisionNet was then trained on this dataset, allowing the network to learn and correct for PSF variations across the entire FOV. This approach enabled robust handling of spatially varying blur, significantly improving the consistency and quality of the reconstructed images. Fine-tuning was performed once per device and required approximately 20 h on a single RTX 4090 GPU.

### Fluorescent Bead Imaging and Resolution Characterization.

To evaluate the imaging performance of PrecisionView, we prepared glass slides coated with 4 µm diameter (Molecular Probes FluoSpheres 4.0 μm, yellow-green fluorescent, 2% solids) and 1 µm diameter fluorescent beads (Fluoresbrite YG Microspheres 1.0 µm, Cat # 17154-10) and performed imaging with PrecisionView and the conventional system for comparison. Beads were imaged at different axial positions ranging from −250 µm to +250 µm from the focal plane with a step size of 50 µm. To quantitatively evaluate resolution, we calculated the FWHM of the intensity profiles of 1 µm beads at various lateral and axial positions for both systems. At each axial depth, 250 µm × 250 µm square ROIs were selected at different spatial locations within the FOV. The FWHM of intensity profiles were calculated along both horizontal and vertical directions for all visible beads within the ROI and averaged to quantify the local lateral spatial resolution. The same process was repeated at all depths for both PrecisionView and the conventional system.

### Animal Specimen and Postmortem Human Specimen Preparation and Imaging.

Freshly resected ex vivo porcine tongue specimens were obtained from a local abattoir. Postmortem human breast tissue was acquired from Accio Biobank Online. A small portion of the tissue was cut with a scalpel and topically stained with 0.01% (w/v) proflavine solution in PBS using a cotton-tipped applicator. The epithelium surface of the porcine tongue and the cut surface of human breast tissue were stained and imaged. Stained specimens were mounted on a glass slide and imaged sequentially using PrecisionView and a conventional configuration with an identical optical layout but without the phase mask. The two systems were mounted side-by-side on a stage at fixed positions and the specimen was imaged through the glass from the bottom surface, where the tissue was in contact with the window. The glass slide containing the specimens was mounted on a three-dimensional translation stage, allowing imaging of different tissue sites across the sample, while the axial stage provided focus adjustment. For cross-system comparison, large-area scans were first obtained using PrecisionView. The same tissue sites were then immediately reimaged with the conventional system by translating the specimen so that the identical locations were positioned within the imaging field of the conventional system without disturbing the tissue. This procedure enabled accurate registration between images acquired by the two systems and direct comparison of corresponding cellular features within the same subregions. The focus was adjusted before imaging each tissue site to ensure that the central imaging region was in focus.

### In Vivo Human Oral Imaging.

The in vivo human study was conducted at Rice University; study participants were adult volunteers aged 18 y or older and without underlying health conditions. The study protocol was approved by the Institutional Review Board (IRB) at Rice University, and written informed consent was obtained from each participant prior to imaging. Before each imaging session, the device underwent a standard high-level disinfection procedure (62). Tissue regions of interest in the oral cavity were stained topically with a 0.01% (w/v) proflavine solution in phosphate-buffered saline, applied using cotton-tipped applicators. Imaging was performed immediately following proflavine application. During imaging, the PrecisionView system was gently placed in contact with various stained tissue sites within the oral cavity. Because the imaging modality was alternated every second, the probe was held stationary at each site for ~2 s to ensure acquisition of sharp, motion-free frames and to facilitate accurate coregistration between nuclear and microvascular images. The probe was then translated by approximately 2 mm to image the adjacent tissue region. Continuous image sequences were acquired throughout the session, and the controlled step size enabled reliable mosaicking during postprocessing. Reconstructed microscopic images showing epithelial cell nuclei and subsurface microvasculature were displayed in real time via the graphical user interface (GUI), while raw image frames were saved for postsession analysis. Each imaging session lasted approximately 1 to 2 min, allowing examination of multiple tissue sites of interest. The acquired raw frames were subsequently processed using PrecisionNet and stitched into composite views using the Image Composite Editor (Microsoft). A total of six healthy volunteers were enrolled in this study. Consistent imaging quality was achieved across all healthy volunteers enrolled in the study.

### Surgical Sample Imaging.

The study was conducted at The University of Texas MD Anderson Cancer Center (MD Anderson), with protocol approval from the IRBs of both MD Anderson and Rice University. Eligible participants included patients aged 18 y or older who were scheduled to undergo LEEP or CKC biopsy for the treatment of cervical dysplasia and had a confirmed negative pregnancy test. Written informed consent was obtained from all participants prior to enrollment. Before each imaging session, the device underwent a standard cleaning and high-level disinfection (62). Immediately following excision, the cervical specimens were imaged using the PrecisionView system. The tissue surface was first cleaned with sterile swabs and saline to remove any residual debris, then stained topically with a 0.01% (w/v) proflavine solution in phosphate-buffered saline, applied using cotton-tipped applicators. During imaging, the PrecisionView system was held by hand and placed in gentle contact with the stained tissue surface, then scanned across the entire specimen. Because the imaging modality was alternated every second, the probe was held stationary at each site for ~2 s to ensure acquisition of sharp, motion-free frames and to facilitate accurate coregistration between nuclear and microvascular images. The probe was then translated by approximately 2 mm to image the adjacent tissue region. Continuous image sequences were acquired throughout the session, and the controlled step size enabled reliable mosaicking during postprocessing. Reconstructed microscopic images revealing epithelial cell nuclei and subsurface microvasculature were displayed in real time via the graphical user interface (GUI), while raw image frames were saved for further analysis. Each imaging session lasted approximately 5 to 8 min, enabling comprehensive examination of the entire excised cervical specimen. After PrecisionView imaging, the cervical specimens were sent to the pathologist within the expected time for histopathological diagnosis per standard of care. The raw image data captured by PrecisionView were subsequently processed using PrecisionNet and stitched into composite views using the Image Composite Editor (Microsoft). Nine patients were enrolled in this study, with consistent imaging quality achieved across all subjects. Data from two patients are shown in Fig. 6 and *SI Appendix*, Fig. S8 and Movie S4. Imaging findings were correlated with final pathology results.

## Supplementary Material

Appendix 01 (PDF)

Movie S1. Real-time video acquisition and reconstruction of PrecisionView at 15 frames per second when imaging the lip mucosa of a healthy volunteer. Microvascular blood flow is clearly observed in several vessels.

Movie S2. Reconstructed video of PrecisionView imaging of the lip mucosa in the oral cavity of a healthy volunteer.

Movie S3. Reconstructed video of PrecisionView imaging of the tongue epithelium in the oral cavity of a healthy volunteer.

Movie S4. Reconstructed video of PrecisionView imaging of the epithelium of ventral tongue in the oral cavity of a healthy volunteer.

Movie S5. Reconstructed video of PrecisionView imaging of a cervix specimen with precancerous lesions.

## Data Availability

The main data supporting the results of this study are available within the paper and its *SI Appendix*. The raw and analyzed datasets generated during the study are available for research purposes from the corresponding author upon request. Custom codes used in this study are available on a GitHub repository (63).
