# Supplementary material for: Deep-learning endomicroscope with large field-of-view and depth-of-field for real-time in vivo imaging of epithelial cancer hallmarks
Source: Proc Natl Acad Sci U S A. 2026 May 11;123(20):e2602705123. doi: 10.1073/pnas.2602705123 (PMC13187819; doi:10.1073/pnas.2602705123)
Supplement: Supplementary file 1 — Appendix 01 (PDF) [file pnas.2602705123.sapp.pdf]

PNAS

Supporting Information for
Deep-learning endomicroscope with large field-of-view and depth-of-field for real-time in vivo imaging of epithelial cancer hallmarks

Huayu Hou, Jimin Wu, Jinyun Liu, Vivek Boominathan, Argaja Shende, Karthik Goli, Jennifer Carns, Richard A. Schwarz, Ann M. Gillenwater, Preetha Ramalingam, Mila P. Salcedo, Kathleen M. Schmeler, Tomasz S. Tkaczyk, Jacob T. Robinson, Ashok Veeraraghavan, and Rebecca R. Richards-Kortum

Ashok Veeraraghavan, Rebecca R. Richards-Kortum
Email: vashok@rice.edu, rkortum@rice.edu.

This PDF file includes:

Supporting text
Figures S1 to S10
Table S1
Legends for Movies S1 to S5
SI References

Other supporting materials for this manuscript include the following:

Movies S1 to S5

Supporting Information Text

Learnable optical layer

In the end-to-end deep neural network, the learnable optical layer simulates the microscope's optical imaging while incorporating passive phase modulation at the Fourier plane. Within this system design, both fluorescence and reflectance imaging in the green spectrum are modeled as incoherent imaging at a wavelength of 530 nm.

To accurately simulate the system, we employed a model based on Fourier optics theory(1, 2). Specifically, the phase mask was placed at the Fourier plane of the imaging system, where the point spread function (PSF) can be expressed as the squared magnitude of the Fourier transform of the pupil function.

The pupil function $P(x_1, y_1, z)$ characterizes the spatial distribution of amplitude and phase modifications introduced to the wavefront at the Fourier plane:

$$P(x_1, y_1, z) = A(x_1, y_1)e^{i\Phi(x_1, y_1, z)} \qquad (S1)$$

The amplitude A is defined by a circular aperture function, representing a disk-shaped aperture with values of unity inside the aperture and zero outside. The phase Φ in this system was modeled as the sum of two distinct components: a defocus component Φ^{DF}, and learnable component representing the phase modulation introduced by the phase mask Φ^{M}. The resulting phase Φ of the pupil function:

$$\Phi(x_1, y_1, z) = \Phi^{DF}(x_1, y_1, z) + \Phi^{M}(x_1, y_1) \qquad (S2)$$

The defocus component Φ^{DF} represents the defocus due to the mismatch between in-focus depth z_0 and the actual imaging depth z. This can be modeled as:

$$\Phi^{DF}(x_1, y_1, z) = k\frac{(x_1^2 + y_1^2)}{2}\frac{z_0 - z}{z_0^2} = kW_m r(x_1, y_1)^2 \qquad (S3)$$

Where $k = \frac{2\pi}{\lambda}$ is the wavenumber and λ is the wavelength, $r(x_1, y_1) = \frac{\sqrt{x_1^2 + y_1^2}}{R}$ represents the normalized radial displacement in the Fourier plane, which is the radial coordinate normalized by the lens aperture radius R, and W_m denotes the maximum optical path-length error at the edge of the pupil caused by defocus.

The phase modulation introduced by the height variation on the phase mask Φ^{M} can be modeled as:

$$\Phi^{M}(x_1, y_1) = k\Delta n\, h(x_1, y_1) \qquad (S4)$$

Where Δn is the refractive index difference between air and the phase mask material, and h represents the height map of the phase mask. The height map h is a learnable parameter within the network and is iteratively updated during the training process.

The PSF in the imaging plane $PSF(x_2, y_2, z)$ can be expressed as the squared magnitude of the Fourier transform $\mathcal{F}\{\cdot\}$ of the pupil function $P(x_1, y_1, z)$:

$$PSF(x_2, y_2, z) = |\mathcal{F}\{P(x_1, y_1, z)\}|^2 \qquad (S5)$$

The image formation process can be described mathematically as the convolution of the object intensity distribution I_0 with the system's PSF:

$$I(x_2, y_2) = \sum_z I_0(x, y, z) * PSF(x_2, y_2, z) \quad (S6)$$

During the phase mask optimization step, we discretized the depth range into multiple layers, each blurred by the corresponding depth-specific PSF. We simulated image formation across a defocus range of −250 μm to +250 μm, discretizing this range into 21 distinct imaging depths. The discrete Fourier transform (DFT) was utilized to model the system's PSF, computed using matrices of 25 × 25 pixels, with the discretization based on the sensor's pixel size. To accelerate convergence during optimization while allowing adequate degrees of freedom, we further constrained the learnable height map by representing it in terms of a finite set of Zernike polynomial basis functions. The height map was parameterized using the first 55 Zernike modes, and this number was selected based on a practical trade-off between model expressivity and stability. This approach was used in prior wavefront modeling studies(3, 4). Specifically, the height map h was constrained as follows:

$$h(x_1, y_1) = \sum_{n=1}^{55} a_n Z_n(x_1, y_1) \quad (S7)$$

Where $Z_n(x_1, y_1)$ is the set of Zernike polynomials, and a_n is the coefficient vector. These coefficients were optimized during end-to-end training. The pupil function was parameterized using the first 55 Zernike modes to model system aberrations. This number was selected to capture low- and mid-order aberrations relevant to the compact optical design while maintaining numerical stability during optimization. Including additional higher-order modes did not yield measurable improvement in reconstruction performance.

Gaussian read noise with standard deviation σ = 0.005 - 0.015 was added to normalized simulated images (intensity range [0, 1]). The noise level was randomly varied during training to improve reconstruction robustness. Under LED-based illumination, speckle effects are negligible, and Gaussian noise provides a reasonable approximation of dominant sensor noise contributions.

Parameter calculation for optical layer

The optical layer in the network is modeled using principles of Fourier optics, and its parameters must be discretized to enable neural network training. Specifically, the following variables must be predefined prior to training:

Δx_0 is the discretized grid size on the object plane. Based on calculation in Eq. S8, in our system, each sensor pixel will image 2.2 μm on the object plane with a 2.2 μm sensor pixel pitch.

$$\Delta x_0 = \delta \frac{f_1}{f_2} = 2.2\mu m \times \frac{13.5mm}{13.5mm} = 2.2\mu m, \quad (S8)$$

where δ is the sensor pixel pitch, f_1 is the back focal length of the objective and f_2 the back focal length of the tube lens.

N is the number of grids on the aperture plane, which we set equal to the number of grids on the object plane. The number must be selected to ensure that the grid on the sensor plane is large

enough to capture the entire PSF at the furthest defocus depth. Based on the calculation in Eq. S9, we selected N = 25 for our system.

$$N \geq \frac{d_{PSF}}{\Delta x_0} = \frac{50\mu m}{2.2\mu m} = 22.72, \qquad (S9)$$

where d_{PSF} is the diameter of the PSF at the furthest defocus depth (250 μm).

Δx_1 is the discretized grid size on the aperture plane, and it is determined by the scaling from the object plane to the Fourier plane.

$$\Delta x_1 = \frac{\lambda f_1}{N \times \Delta x_0} = \frac{530nm \times 13.5mm}{25 \times 2.2\mu m} = 130.1\mu m, \qquad (S10)$$

n is the number of discretized grids of the phase mask, which must ensure that the features of the phase mask cover the entire back pupil.

$$n = \frac{d_{pupil}}{\Delta x_1} = \frac{3mm}{130.1\mu m} \approx 23, \qquad (S11)$$

where d_{pupil} is the diameter of the object back pupil. In our system, we have a back pupil size of 3 mm.

PrecisionNet

Recovering an all-in-focus image from a blurry measurement without prior defocus information represents a blind deblurring problem, optimally addressed using deep neural networks. To solve this, we developed a deep neural network, we referred to as PrecisionNet, designed specifically for the image reconstruction task. PrecisionNet employs a modified U-Net architecture(5) that leverages multi-scale feature extraction with skip connections between corresponding encoder and decoder blocks(6–8). At each scale, PrecisionNet incorporates an additional residual block consisting of two convolutional layers with kernel size 3×3. Additionally, a pyramid pooling module (PPM)(9) is integrated at the bottleneck layer to effectively capture global contextual information. The encoder utilizes pixel-shuffle convolution(10) for downsampling, whereas the decoder employs bilinear interpolation for upsampling. The detailed architecture of PrecisionNet is illustrated in Fig. S5. For memory-efficient training, PrecisionNet takes blurry input images sized 368×480 pixels and outputs the corresponding sharp, in-focus images. Once trained, PrecisionNet can reconstruct images directly at the raw capture resolution (1960×2592 pixels).

Training dataset and implementation

To fully leverage the power of deep learning, a large and diverse set of training images that capture a wide range of imaging features is essential. However, collecting large-scale, paired datasets for lensless imaging systems remains a significant challenge, as supervised training typically relies on well-labeled ground truth data. To address this, we employed a forward simulator to generate synthetic measurements from diverse high-resolution image sources. These includes open-source microscopy datasets(11–13), spanning multiple modalities (bright-field, confocal, and two-photon fluorescence), histopathology images(1), widefield fluorescence images of standard microscope slides acquired with a 4× (Nikon Fluor) objective(14), proflavine-stained oral cancer resections imaged with a 10× (RMS10X) objective(1), and widefield microscopy data containing vascular features(15). Specifically, we selected 1,000 images from the open-source microscopy datasets, 600 histopathology images of healthy and cancerous human tissues (brain, lungs, mouth, colon, cervix, and breast) from The Cancer Genome Atlas

(TCGA) FFPE slides, and 800 widefield microscopy images captured in-house for the training process. The training dataset includes microscopy images from both fluorescence and reflectance imaging modalities to optimize the dual-modality imaging of PrecisionView. From the combined dataset, we randomly selected 2,000 images for training, 200 for validation, and reserved another 200 for testing. During training, images were randomly cropped and augmented with rotations, flips, and brightness adjustments to improve model generalization.

Appropriate loss functions are critical for ensuring high-quality image reconstructions. The loss function used to train the end-to-end network was a weighted combination of pixel-wise L1 and L2 losses (Eq. S12), computed between the reconstructed all-in-focus image stack and the corresponding ground truth images:

$$\mathcal{L} = \lambda_1 L_1 + \lambda_2 L_2, \qquad (S12)$$

where we used $\lambda_1 = 0.2$ and $\lambda_2 = 1$ for the training.

The network was trained using the Adam optimizer(16), with a learning rate of 1e-9 for the optical layer and 1e-4 for the PrecisionNet. Training and testing were accelerated using a graphics processing unit (GPU). Specifically, model training was performed on a single GPU (Nvidia GeForce RTX 4090 GPU, 24 GB memory), requiring approximately 24 hours to complete 100 epochs with a batch size of 20. After training, the network achieved a reconstruction time of approximately 0.025 s per 8-bit frame at a resolution of 1960 × 2592 pixels on the same GPU. A custom user interface was developed to enable real-time visualization of both raw image captures and reconstructed output from the PrecisionView system. The interface supports real-time display of reconstructed video streams at a frame rate of up to 22 FPS on the training workstation, providing responsive feedback during imaging sessions. The user interface and trained reconstruction model were deployed on a laptop (Dell Mobile Precision with an Intel i9-11950H 8-core CPU and NVIDIA GeForce RTX 3080 GPU), enabling raw image acquisition at up to 30 FPS and real-time reconstruction at up to 7 FPS.

Phase mask fabrication

The phase mask was fabricated using a 3D maskless two-photon photolithography system (Nanoscribe, Quantum X) in high-resolution dip-in liquid lithography mode (Fig. S6). The mask was fabricated on a 700 µm thick fused silica substrate using the photoresist resin IP-S. The two-photon grayscale lithography enabled a smooth surface quality, while the adaptive voxel size could be tuned based on laser power variations, with a maximum power of 50 mW, a slicing distance of 1 µm, and a hatching distance of 0.2 µm. After exposure, the fabricated mask was immersed in SU-8 developer for 15 minutes, followed by a 2-minute soak in isopropyl alcohol. The substrate was laser cut to 12 mm diameter to fit the design of the housing and fitted with an opaque mask to create an aperture containing only the phase mask.

Image quality quantification

Image quality was quantified for both the conventional system and PrevisionView using signal-to-noise ratio (SNR), signal-to-background ratio (SBR), and contrast. Regions of interest (ROIs)

corresponding to nuclei (or vessels) were manually selected as signal regions, while adjacent non-structural tissue areas were selected as background regions.

The mean signal intensity (μ_{signal}), mean background intensity ($\mu_{\text{background}}$), and background standard deviation ($\sigma_{\text{background}}$) were computed from the ROIs. SNR, SBR and contrast were calculated as:

$$SNR = \frac{\mu_{\text{signal}} - \mu_{\text{background}}}{\sigma_{\text{background}}} \quad (S13)$$

$$SBR = \frac{\mu_{\text{signal}}}{\mu_{\text{background}}} \quad (S14)$$

$$contrast = \frac{\mu_{\text{signal}} - \mu_{\text{background}}}{\mu_{\text{background}}} \quad (S15)$$

Metrics were computed across 20 ROIs per each system condition for comparison (Fig. S10).

Image stitching

Large-area dual-modality images were generated from reconstructed PrecisionView images acquired during manual scanning of tissue. Frames with minimal motion blur and clear visualization of nuclear and vascular features were manually selected. Paired nuclear and vascular images with precise co-registration and minimal spatial shift at each tissue site were included in the stitching process. Adjacent imaging sites were separated by ~2 mm, resulting in greater than 50% interframe overlap for the selected frames. The selected frames were stitched using the Image Composite Editor (Microsoft) software. Nuclear and vascular image sequences were stitched independently to generate separate large-area mosaics. The stitched images shown in this manuscript were downsampled by 8× for visualization purposes.

Figures

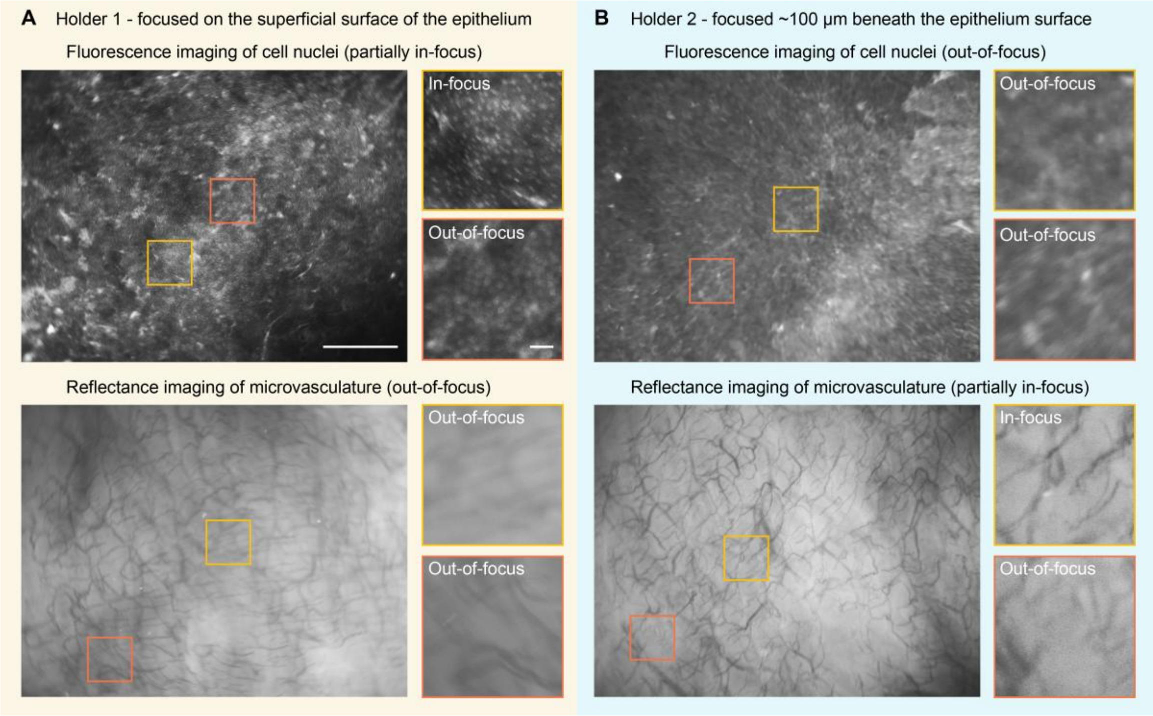

Fig. S1. In vivo imaging of lip mucosa in healthy volunteers using a conventional device at different imaging depths. (*A*) Representative frames of cell nuclei and microvasculature captured using the conventional device with a holder that maintains focus on the superficial surface of the epithelium. At this focal plane, cell nuclei appear partially in focus, with out-of-focus regions primarily resulting from tissue surface variation. Microvasculature appears mostly out of focus due to focal plane mismatch. Scale bar, 1 mm. Zoom-ins highlight an in-focus region and a corresponding out-of-focus region of cell nuclei, and two out-of-focus regions of microvasculature. Scale bar, 100 μm. (*B*) Representative frames of cell nuclei and microvasculature captured using the conventional device with a holder that maintains focus ~100 μm beneath the epithelium surface. At this focal plane, microvasculature appears partially in focus, with out-of-focus regions primarily resulting from tissue surface variation and microvasculature depth variation. Cell nuclei appear mostly out of focus due to focal plane mismatch. Zoom-ins highlight an in-focus region and a corresponding out-of-focus region of microvasculature, and two out-of-focus regions of cell nuclei.

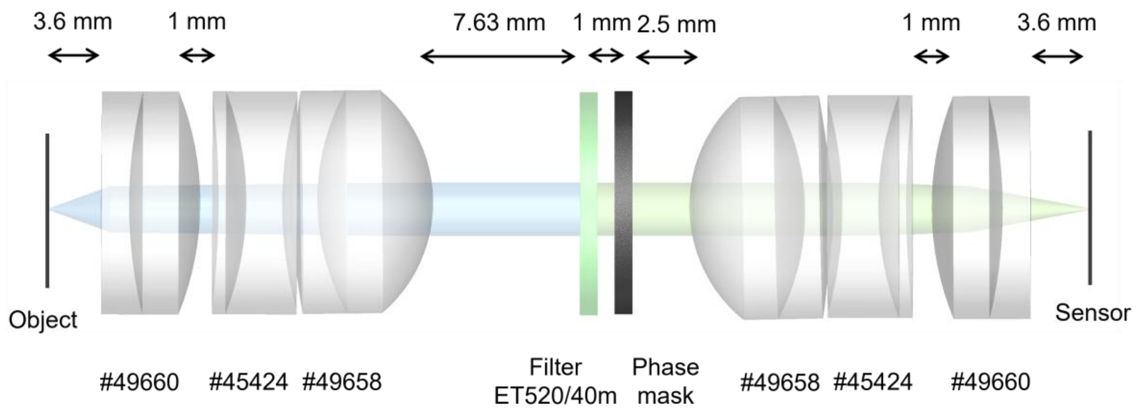

Fig. S2. Optical diagram of PrecisionView. The optical system of PrecisionView consists of two subassemblies of optics, an emission filter (Chroma ET520/40m), and a phase mask, all mounted in 3D-printed housings (Formlabs Form 3+, Black Resin). The objective subassembly contains three achromatic lenses (#45-424, #49-658, #49-660, Edmund Optics), and the tube lens subassembly is identical to the objective lens and placed immediately after the emission filter and phase mask.

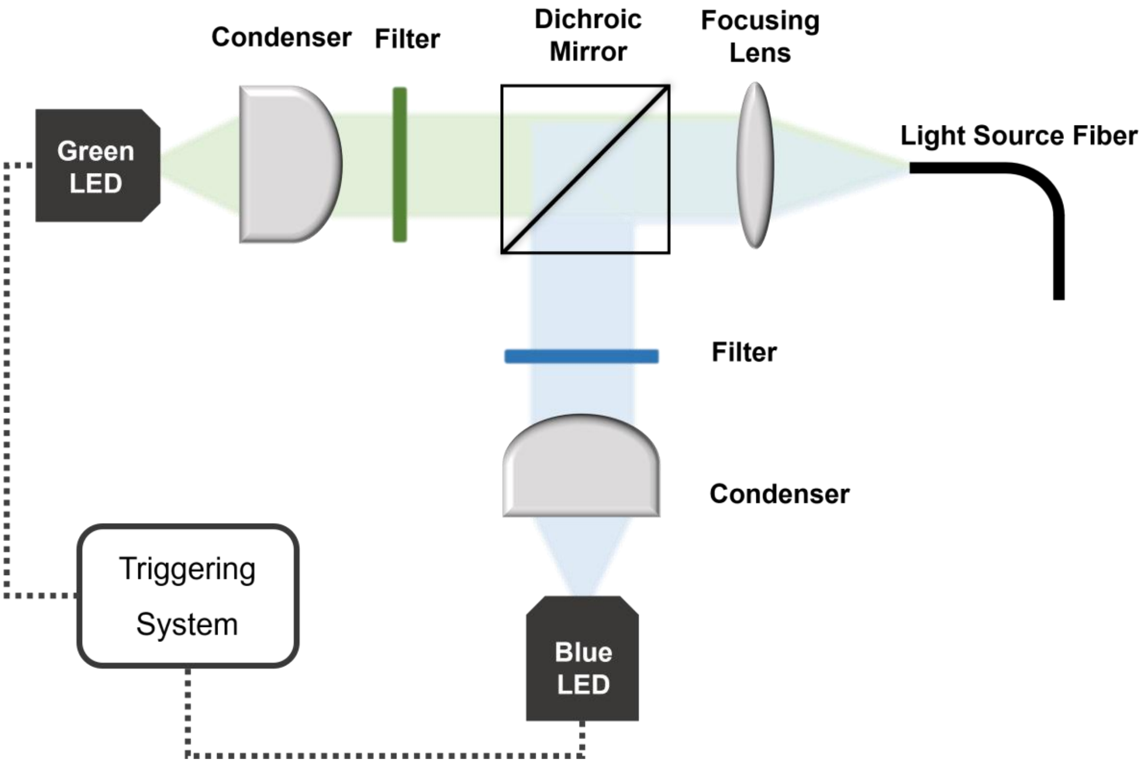

Fig. S3. Optical diagram of the dual-wavelength illumination module for PrecisionView. All fibers used for PrecisionView illumination are bundled and coupled to an LED-based illumination module. The illumination module contains a blue LED (Mouser LZ4-40B208-0000) with a condenser and an excitation filter (#84-705, 475 nm Shortpass Filter, Edmund Optics), a green LED (Mouser LZ4-40G108-0000_G2) with a condenser and a bandpass filter (Chroma ET520/40m), and a dichroic mirror (Thorlabs DMLP490R, 490 nm Longpass) to enable switching between blue and green illumination. An Arduino (UNO R3) was used to control the synchronization of the illumination.

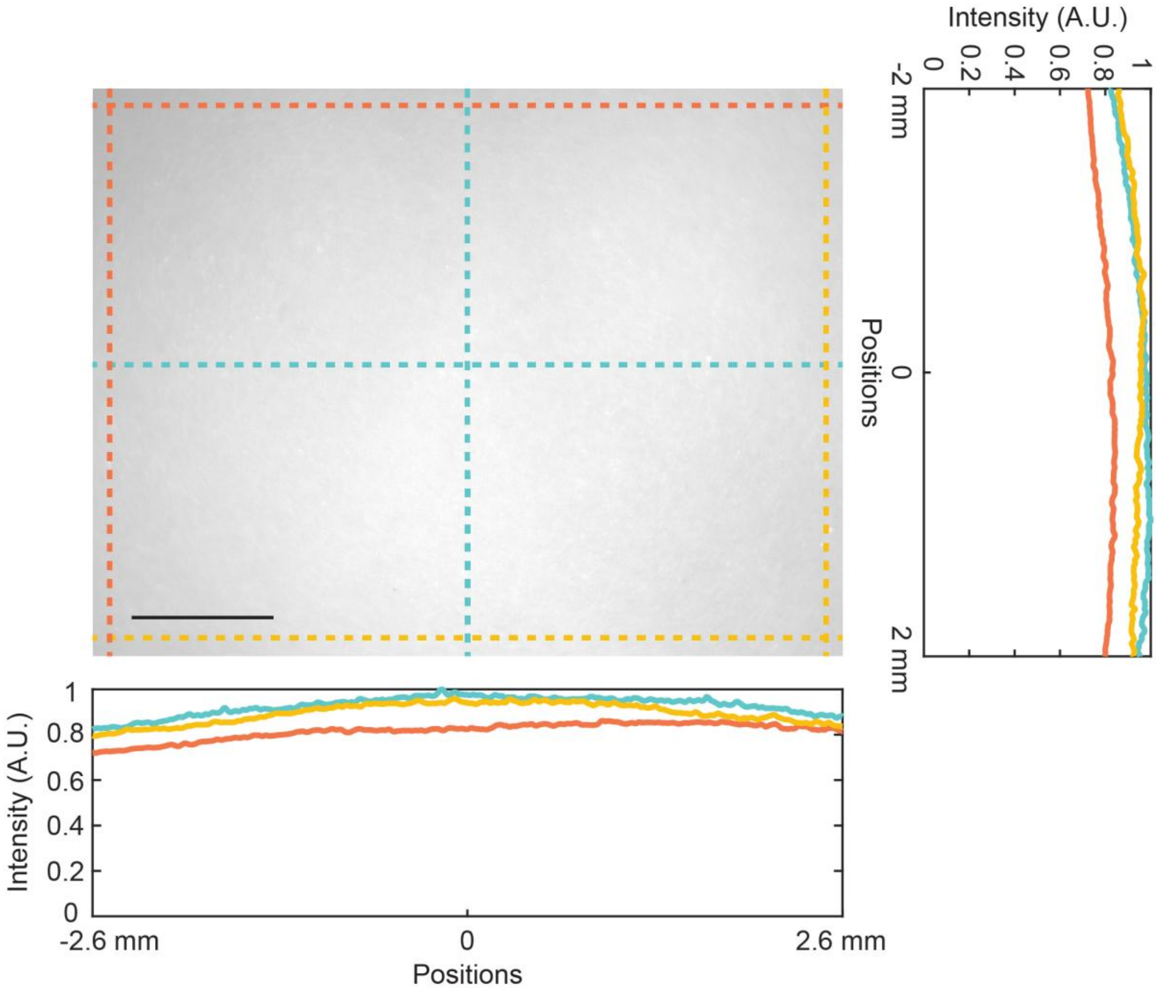

Fig. S4. PrecisionView illumination profile. Experimental capture of the illumination pattern of PrecisionView by imaging a fluorescent phantom at designed working distance. Uniform illumination is achieved over the entire 5.2 mm × 3.9 mm field-of-view. Scale bar, 1 mm.

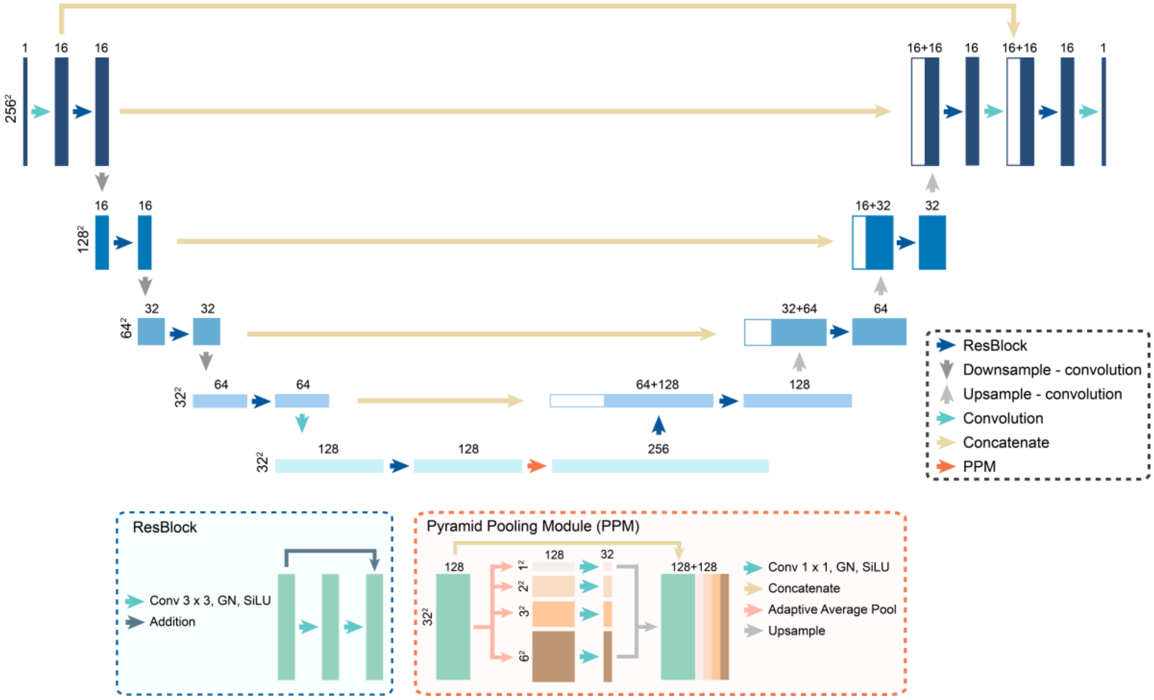

Fig. S5. PrecisionNet structure. PresicionNet is based on a modified U-Net architecture that leverages multi-scale feature extraction with skip connections between corresponding encoder and decoder blocks. At each scale, PrecisionNet incorporates an additional residual block consisting of two convolutional layers with kernel size 3×3. Additionally, a pyramid pooling module (PPM) is integrated at the bottleneck layer to effectively capture global contextual information. The encoder utilizes pixel-shuffle convolution for downsampling, whereas the decoder employs bilinear interpolation for upsampling.

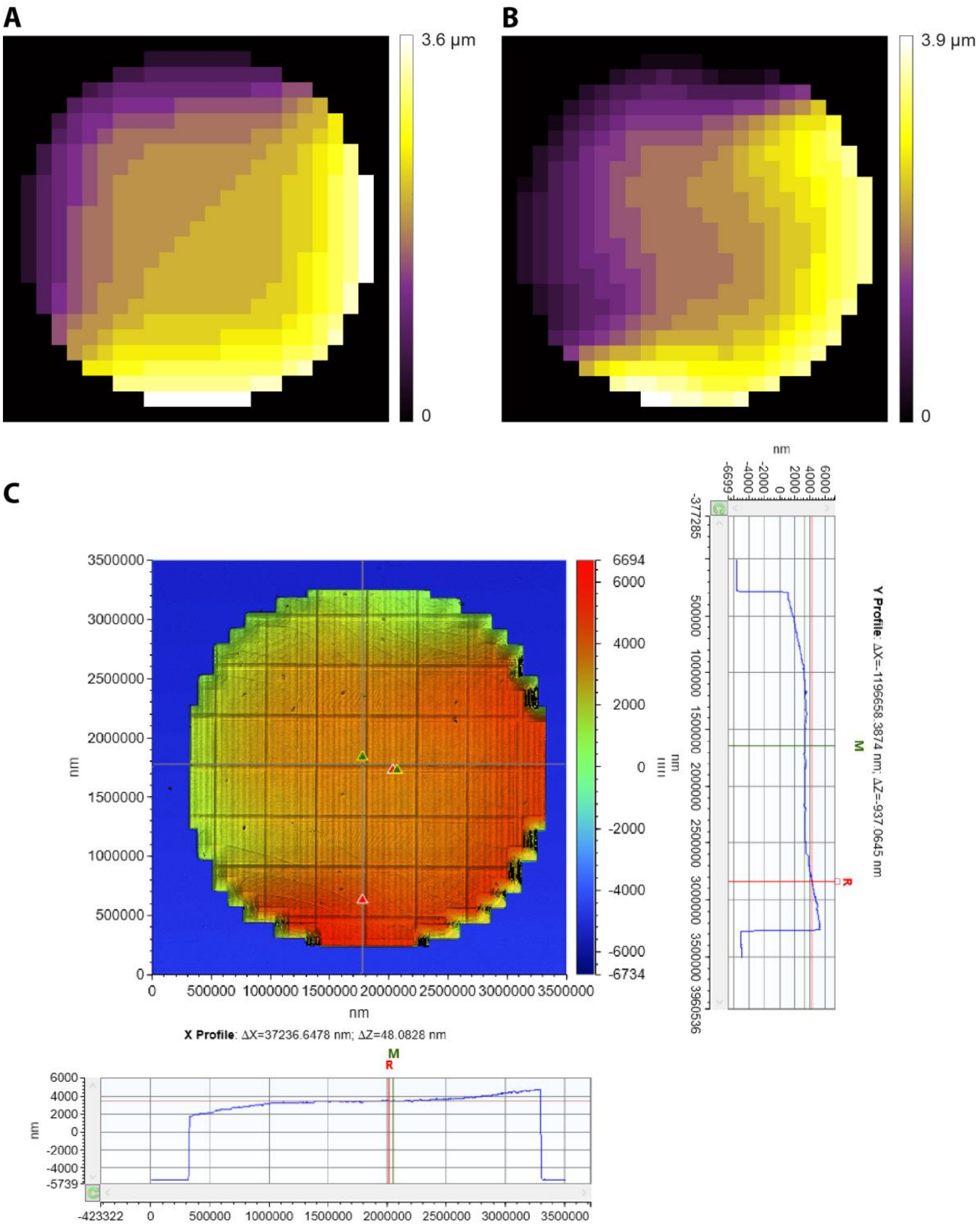

Fig. S6. Phase mask design and fabrication. (*A*) Initialization height map of the phase mask using a Cubic phase mask pattern. (*B*) Phase mask height map after the end-to-end training. (*C*) Height map of the fabricated phase mask measured by optical profiler.

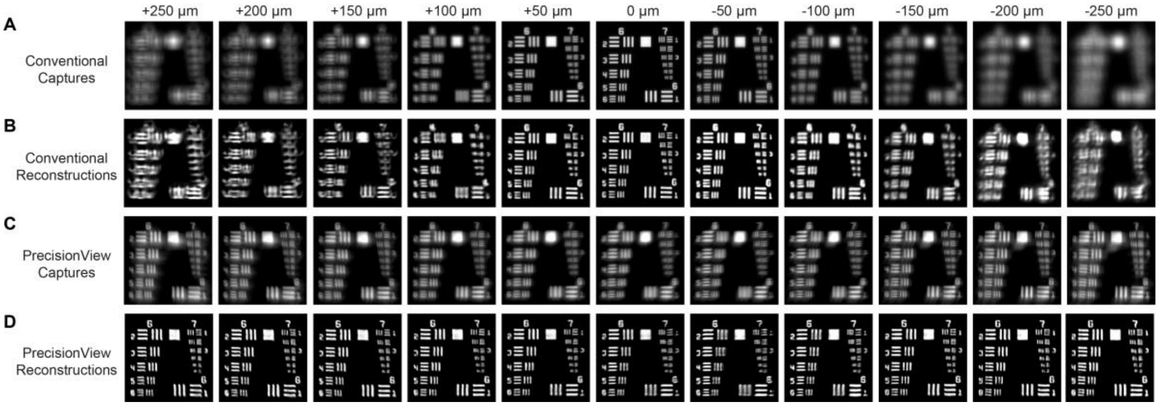

Fig. S7. Comparison of optical and computational contributions to PrecisionView using a USAF resolution target. (*A*) Captured images using the conventional system (no phase mask, no reconstruction) at different depths. (*B*) PrecisionNet reconstructions applied to conventional system captures (reconstruction algorithm only). The network was trained using PSFs measured from the conventional system with the same training strategy used for PrecisionView. (*C*) Captured images using PrecisionView optics incorporating the engineered phase mask (phase mask only, no reconstruction) at different depths. (*D*) PrecisionNet reconstructions applied to PrecisionView captures (full system: phase mask + reconstruction). These results show that neither deep-learning–based reconstruction alone nor phase-mask-enabled wavefront shaping alone is sufficient to effectively extend the DOF. In contrast, the end-to-end optimization implemented in PrecisionView enables DOF extension and overall imaging performance improvement.

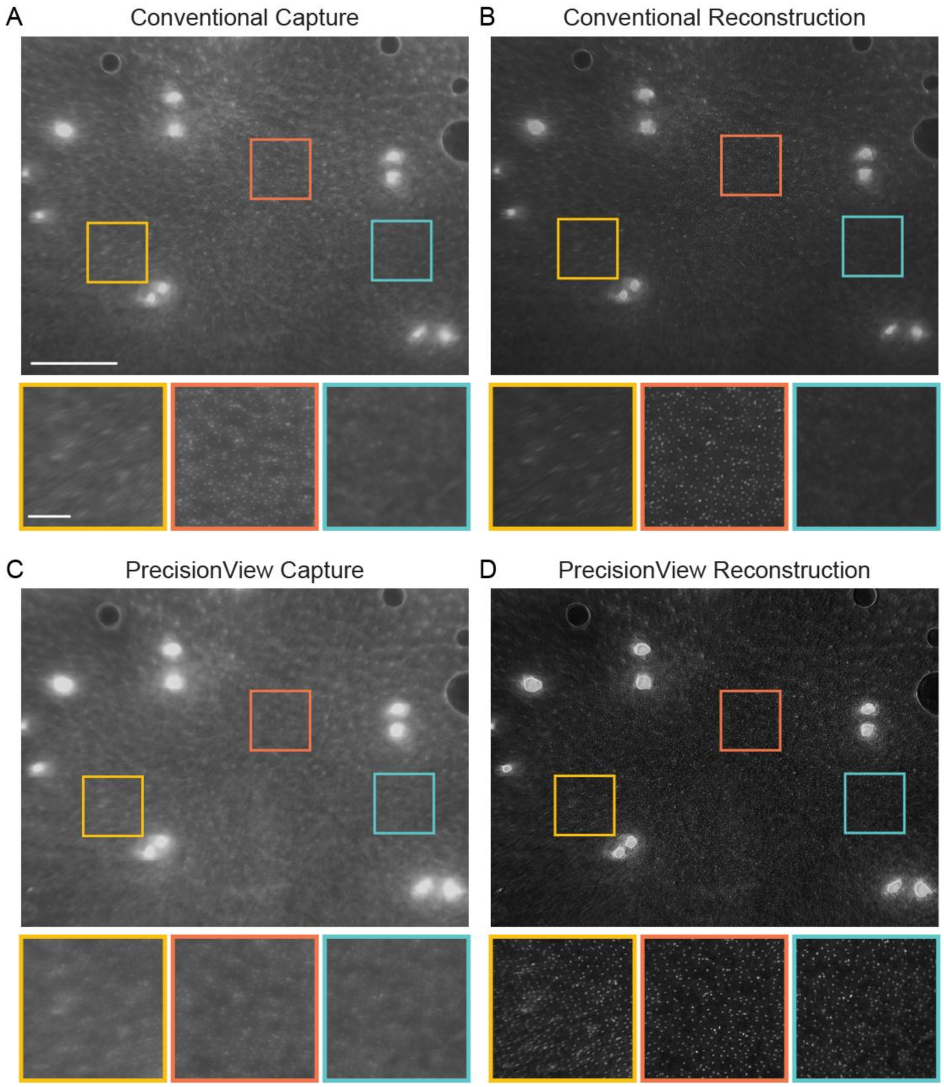

Fig. S8. Comparison of conventional imaging and PrecisionView configurations using a porcine tongue specimen. (*A*) Captured image using the conventional system (no phase mask, no reconstruction). (*B*) PrecisionNet reconstruction applied to conventional system capture (reconstruction algorithm only). The network was trained using PSFs measured from the conventional system with the same training strategy used for PrecisionView. (*C*) Captured image using PrecisionView optics incorporating the engineered phase mask (phase mask only, no reconstruction). (*D*) PrecisionNet reconstruction applied to PrecisionView capture (full system: phase mask + reconstruction). Scale bar: full image, 1 mm, zoom-in views, 200 μm. Imaging results from real tissue specimens further validate that the enhanced imaging performance resolving nuclei across the full FOV is achievable only through the integrated, end-to-end optimization framework.

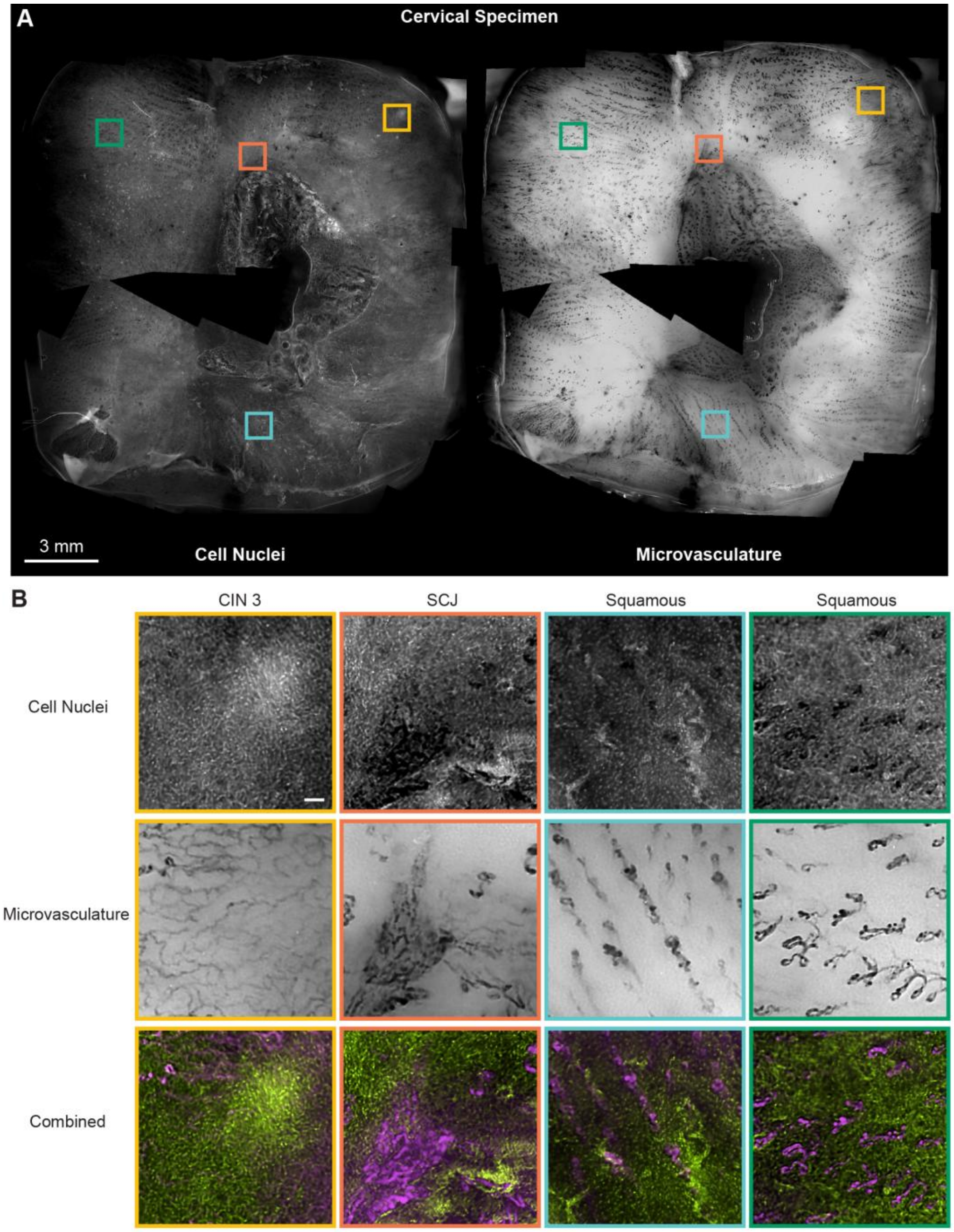

Fig. S9. Additional ex vivo co-registered maps of cell nuclei and microvasculature from a freshly resected cervical specimen with precancerous lesions. (*A*) Stitched dual-modality maps of cell nuclei and microvasculature from the epithelial surface of a freshly resected cervical specimen, covering a tissue area of ~ 3.5 cm^2. Video acquisition time, ~ 7 min. Scale bar of stitched maps, 3 mm. (*B*) Zoom-ins of annotated ROIs in panel A, showing separate and combined high-resolution images of cell nuclei and microvasculature for each ROI. Scale bars, 100 μm. Images of normal squamous epithelium show uniformly shaped small nuclei underlying normal capillary loops. Images from the squamocolumnar junction (SCJ) show smaller, more densely packed nuclei. In contrast, the image acquired from a region with CIN3, shows enlarged, pleomorphic nuclei and increased subsurface vessel density.

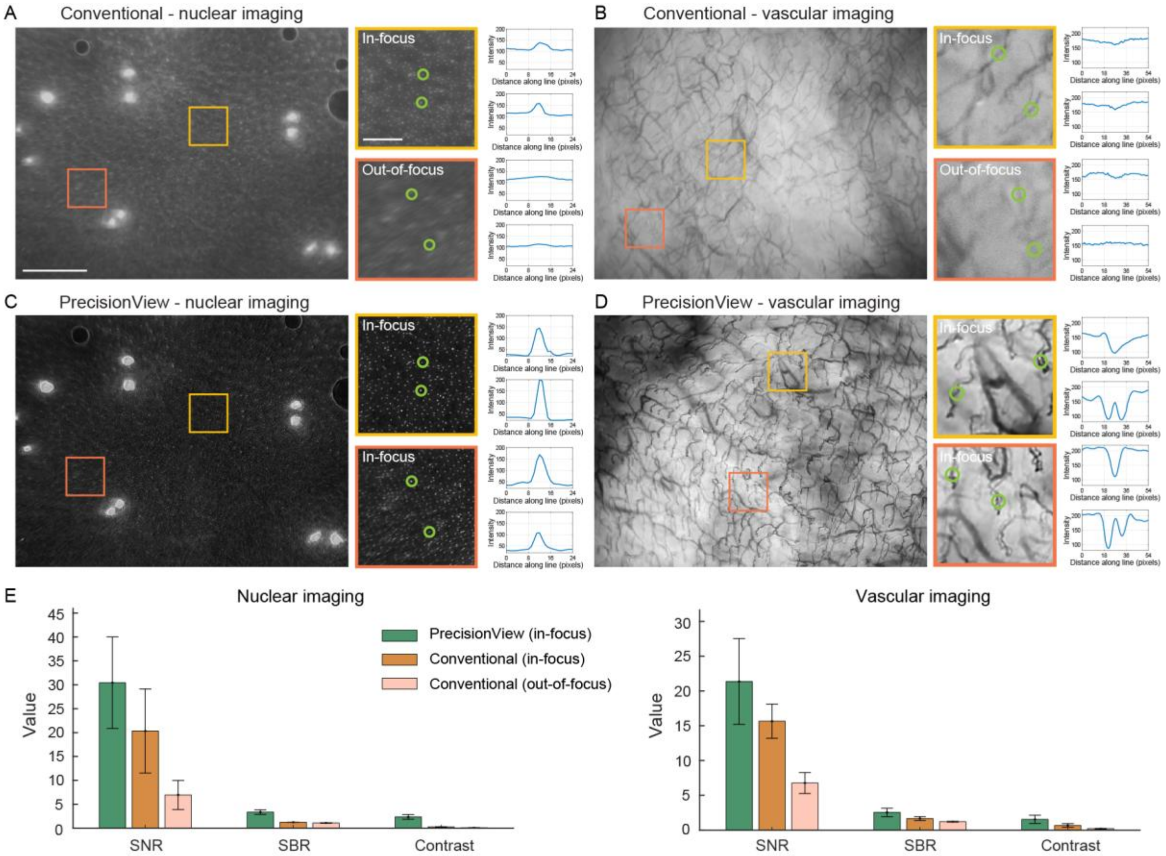

Fig. S10. Representative comparison of nuclear and vascular image contrast between the conventional system and PrecisionView. (*A*) Representative fluorescence images of cell nuclei acquired using the conventional system (in-focus and out-of-focus). (*B*) Representative reflectance images of microvasculature acquired using the conventional system (in-focus and out-of-focus). (*C*) Representative fluorescence images of cell nuclei acquired using PrecisionView (in-focus). (*D*) Representative reflectance images of microvasculature acquired using PrecisionView (in-focus). Line intensity profiles across selected nuclei and vessels (green circle) are shown to illustrate contrast differences between systems. PrecisionView demonstrates improved contrast and preservation of structural features compared with the conventional configuration. Scale bar: full image, 1 mm, zoom-in views, 200 µm. (*E*) Bar plots show the mean and standard deviation of SNR, SBR, and contrast for PrecisionView and the conventional system (in-focus and out-of-focus) in nuclear imaging. (*F*) Bar plots show the mean and standard deviation of SNR, SBR, and contrast for PrecisionView and the conventional system (in-focus and out-of-focus) in vascular imaging.

Tables

Table S1. Parts list and cost breakdown of PrecisionView, excluding the computer and GPU required for reconstruction

Item	Part number	Vendor	Price	Quantity	Cost
**PrecisionView**					**Total cost: $2,105**
Achromatic Lenses	45-424	Edmund Optics	$82	2	$164
Achromatic lenses	49-658	Edmund Optics	$143	2	$286
Achromatic lenses	49-660	Edmund Optics	$143	2	$286
Camera	MU050MR-SY	XIMEA	$870	1	$870
Phase mask	NA	Fabricated in house	$30	1	$30
Green bandpass filter	ET520/40m	Chroma	$365	1	$365
Illumination fibers	02-532	Edmund Optics	$1.5/ft	20 ft	$30
Sapphire window	20-633	Edmund Optics	$74	1	$74
**Light source**					**Total cost: $994**
Blue LED	LZ4-40B208-0000	Mouser	$13	1	$13
Green LED	LZ4-40G108-0000_G2	Mouser	$15	1	$15
Blue excitation filter	84-705	Edmund Optics	$276	1	$276
Green bandpass filter	ET520/40m	Chroma	$365	1	$365
Dichroic mirror	DMLP490R	Thorlabs	$298	1	$298
Arduino	UNO R3	Arduino	$27	1	$27
					**Total cost: $3,099**

Movie S1 (separate file). Real-time video acquisition and reconstruction of PrecisionView at 15 frames per second when imaging the lip mucosa of a healthy volunteer. Microvascular blood flow is clearly observed in several vessels.

Movie S2 (separate file). Reconstructed video of PrecisionView imaging of the lip mucosa in the oral cavity of a healthy volunteer.

Movie S3 (separate file). Reconstructed video of PrecisionView imaging of the tongue epithelium in the oral cavity of a healthy volunteer.

Movie S4 (separate file). Reconstructed video of PrecisionView imaging of the epithelium of ventral tongue in the oral cavity of a healthy volunteer.

Movie S5 (separate file). Reconstructed video of PrecisionView imaging of a cervix specimen with precancerous lesions.

SI References

1. L. Jin, et al., Deep learning extended depth-of-field microscope for fast and slide-free histology. Proc. Natl. Acad. Sci. U.S.A. 117, 33051–33060 (2020).
2. L. Jin, et al., DeepDOF-SE: affordable deep-learning microscopy platform for slide-free histology. Nat Commun 15, 2935 (2024).
3. Y. Wu, V. Boominathan, H. Chen, A. Sankaranarayanan, A. Veeraraghavan, PhaseCam3D — Learning Phase Masks for Passive Single View Depth Estimation in 2019 IEEE International Conference on Computational Photography (ICCP), (IEEE, 2019), pp. 1–12.
4. Y. Shechtman, S. J. Sahl, A. S. Backer, W. E. Moerner, Optimal Point Spread Function Design for 3D Imaging. Phys. Rev. Lett. 113, 133902 (2014).
5. O. Ronneberger, P. Fischer, T. Brox, “U-Net: Convolutional Networks for Biomedical Image Segmentation” in Medical Image Computing and Computer-Assisted Intervention – MICCAI 2015, Lecture Notes in Computer Science., N. Navab, J. Hornegger, W. M. Wells, A. F. Frangi, Eds. (Springer International Publishing, 2015), pp. 234–241.
6. K. He, X. Zhang, S. Ren, J. Sun, Deep Residual Learning for Image Recognition in 2016 IEEE Conference on Computer Vision and Pattern Recognition (CVPR), (IEEE, 2016), pp. 770–778.
7. Z. Zhang, Q. Liu, Y. Wang, Road Extraction by Deep Residual U-Net. IEEE Geosci. Remote Sensing Lett. 15, 749–753 (2018).
8. B. Ghanekar, et al., Passive Snapshot Coded Aperture Dual-Pixel RGB-D Imaging in 2024 IEEE/CVF Conference on Computer Vision and Pattern Recognition (CVPR), (IEEE, 2024), pp. 25348–25357.
9. H. Zhao, J. Shi, X. Qi, X. Wang, J. Jia, Pyramid Scene Parsing Network in 2017 IEEE Conference on Computer Vision and Pattern Recognition (CVPR), (IEEE, 2017), pp. 6230–6239.
10. W. Shi, et al., Real-Time Single Image and Video Super-Resolution Using an Efficient Sub-Pixel Convolutional Neural Network in 2016 IEEE Conference on Computer Vision and Pattern Recognition (CVPR), (IEEE, 2016), pp. 1874–1883.
11. V. Ljosa, K. L. Sokolnicki, A. E. Carpenter, Annotated high-throughput microscopy image sets for validation. Nat Methods 9, 637–637 (2012).
12. Y. Zhang, et al., A Poisson-Gaussian Denoising Dataset With Real Fluorescence Microscopy Images in 2019 IEEE/CVF Conference on Computer Vision and Pattern Recognition (CVPR), (IEEE, 2019), pp. 11702–11710.

13. S. Arslan, T. Ersahin, R. Cetin-Atalay, C. Gunduz-Demir, Attributed Relational Graphs for Cell Nucleus Segmentation in Fluorescence Microscopy Images. IEEE Trans. Med. Imaging 32, 1121–1131 (2013).
14. J. Wu, V. Boominathan, A. Veeraraghavan, J. T. Robinson, Real-time, deep-learning aided lensless microscope. Biomed. Opt. Express 14, 4037 (2023).
15. J. Wu, Y. Chen, A. Veeraraghavan, E. Seidemann, J. T. Robinson, Mesoscopic calcium imaging in a head-unrestrained male non-human primate using a lensless microscope. Nat Commun 15, 1271 (2024).
16. D. P. Kingma, J. Ba, Adam: A Method for Stochastic Optimization. [Preprint] (2017). Available at: http://arxiv.org/abs/1412.6980 [Accessed 7 April 2026].
